# Promiscuous and multivalent interactions between Eps15 and partner protein Dab2 generate a complex interaction network

**DOI:** 10.1038/s41467-025-63090-1

**Published:** 2025-08-21

**Authors:** Andromachi Papagiannoula, Ida Marie Vedel, Kathrin Motzny, Maud Tengo, Arbesa Saiti, Sigrid Milles

**Affiliations:** 1 Leibniz-Forschungsinstitut für Molekulare Pharmakologie, Robert-Rössle-Straße 10, Berlin, Germany; 2 Freie Universität Berlin, Department of Biology, Chemistry, Pharmacy, Berlin, Germany; 3 Université Grenoble Alpes, CNRS, CEA, IBS, Grenoble, France

**Keywords:** Intrinsically disordered proteins, Endocytosis, Solution-state NMR

## Abstract

Clathrin-mediated endocytosis depends on complex protein interactions. Eps15 plays a key role through interactions of its three EH domains with Asn-Pro-Phe (NPF) motifs in intrinsically disordered regions (IDRs) of other endocytic proteins. Using nuclear magnetic resonance spectroscopy, we investigate the interaction between Eps15’s EH domains and a highly disordered Dab2 fragment (Dab2_320-495_). We find that the EH domains exhibit binding promiscuity, recognizing not only the NPF motif of Dab2 but also other phenylalanine containing motifs. This promiscuity enables interactions with Eps15’s own IDR (Eps15_IDR_), which lacks NPF motifs, suggesting a self-inhibitory state that promotes liquid-liquid phase separation. Despite competing for the same EH domain binding sites, Eps15_IDR_ and Dab2_320-495_ can bind EH123 simultaneously, forming a highly dynamic interaction network that facilitates the recruitment of Dab2_320-495_ into Eps15 condensates. Our findings provide molecular insights into the competitive interactions shaping the early stages of clathrin-mediated endocytosis.

## Abstract (web summary)

Eps15, a protein involved in endocytosis initiation, uses small folded EH domains to bind disordered partner proteins and its own disordered tail, thus forming a dynamic network via phase separation that may help organize early endocytic events.

## Introduction

**Fig. 1:**
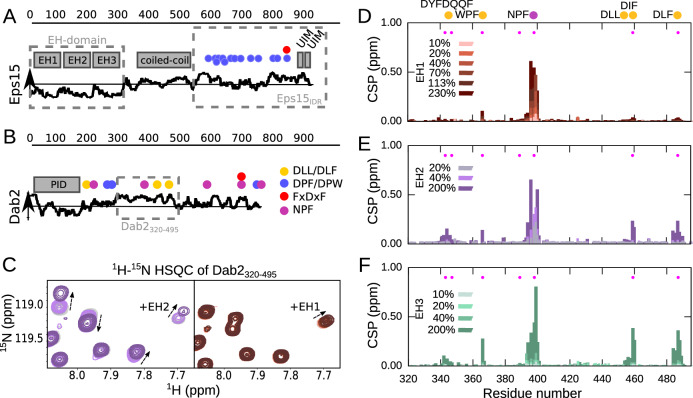
Interaction between Dab2_320–495_ and the individual EH domains of Eps15. Schematic illustration of **(A)** Eps15 and **(B)** Dab2. The disorder prediction (IUPred2A^23^) is shown as a black curve along the sequence. Above horizontal line: predicted disordered, Below horizontal line: predicted ordered. The folded domains are shown as gray boxes above the disorder prediction. Putative interaction motifs are shown as colored circles along the sequence (legend on the right of Dab2). DLL/DLF: clathrin binding; DPF/DPW: AP2α binding; FxDxF: AP2β binding; NPF: EH domain binding. The stretches used in this study are boxed with gray dashed lines. **C** Zoom into a ^1^H−^15^N HSQC spectrum of Dab2_320–495_ in the absence and presence of EH2 (left) and EH1 (right). **D–F** CSPs of 100 μM Dab2_320–495_ in the presence versus the absence of increasing concentrations of EH1 (D), EH2 (E) and EH3 (F), respectively. Color legends are displayed in the respective plots. Filled pink circles denote positions of phenylalanines.

Clathrin-mediated endocytosis (CME) is the major pathway for cargo uptake into the eukaryotic cell, often with transmembrane receptors as cargoes. The important cellular process requires a complex network of interactions, which finally results in the formation and uptake of a clathrin-coated vesicle. During the early phases of the process that takes roughly one to two minutes^1^, intrinsically disordered protein regions (IDRs) of the endocytic initiators play a major role^2^. While folded proteins mainly exist in one stable conformation, IDRs interconvert rapidly between many different conformations due to their relatively flat energy landscape. IDRs often recognize their folded partner proteins through small interaction regions of only a few residues, also called linear motifs^3,4^. This is also the case in CME, where IDRs of endocytic initiators interact with, for example, the major adapter protein complex AP2 or clathrin^5–9^, allowing them to establish a robust multivalent interaction network which allows for rapid rearrangement of the different protein members. Eps15, together with FCHo1/2 proteins, counts among the first proteins to arrive at the endocytic pit^10^. It has three N-terminal Eps15 homology domains (EH domains), which share the common fold of two helix-loop-helix motifs (EF-hands) spaced by a short β-sheet^11^. The EH domains are followed by a central coiled-coil domain, through which Eps15 dimerizes^12,13^, and a large IDR of around 400 residues in length (Fig. 1A)^14^. Eps15 tetramers, formed by two anti-parallel Eps15 dimers, have also been observed, seemingly requiring interaction between the EH domains and the IDR from opposite dimers^13^. FCHo1/2, which also forms dimers, uses its C-terminal µ homology domain (µHD) to interact with the multiple Asp-Pro-Phe (DPF) motifs contained in Eps15’s IDR^15^. The multivalent interaction network between Eps15 and FCHo1/2 enables the formation of liquid-like protein droplets, which has been proposed as a process to catalyze the early steps of CME^16^. The EH domains of Eps15, for example, can bind to Asn-Pro-Phe (NPF) motif containing proteins^17^, such as Epsin1, Stonin2^18^ or Dab2 (disabled homolog 2)^19^. While interactions between EH domains and both Epsin1 and Stonin2 have been characterized at molecular detail, the nature of Dab2’s interaction with EH domains remains to date elusive. Dab2 has an N-terminal folded phosphotyrosine interacting domain (PID), which it uses to anchor itself to the membrane and transmembrane cargo, followed by a large IDR of around 500 residues in length, which comprises both DPF as well as NPF motifs (Fig. 1B)^20^. Both Eps15 and Dab2 belong to the class of clathrin-associated sorting proteins (CLASPs), which regulate cargo sorting during the early phases of CME and usually rely on downstream factors, such as the major adaptor protein complex AP2 or clathrin^3,21,22^. Dab2 has special properties as a CLASP since it can lead to successful CME of specific cargoes even in the absence of AP2. These cargoes, for example, integrin β1, are specifically recognized by the Dab2 PID. Importantly, binding of Eps15 to Dab2 is required for the AP2-independent internalization of these protein cargoes^19^, highlighting the importance of understanding the communication between Eps15 and Dab2.

Comprehending an interaction between two proteins that comprise a significant content of IDRs is challenging. In this work, we therefore chose to use nuclear magnetic resonance (NMR) spectroscopy, which is the only experimental technique able to provide amino-acid resolution of such dynamic protein systems. Using NMR spectroscopy, we thus investigated the region of Dab2 that is predicted to be most disordered, residues 320–495 (Dab2_320–495_), its conformational sampling, as well as its interactions with the EH domains of Eps15. All EH domains interact preferentially with the only NPF motif within Dab2_320–495_, but EH2 and EH3 demonstrate a high level of binding promiscuity towards other phenylalanine-containing motifs. This promiscuity is maintained for the combined EH domains, comprising EH1, EH2 and EH3 (EH123), and surprisingly enables Eps15’s own IDR (Eps15_IDR_), which does not possess any NPF motifs, to bind to EH123 and thereby partially occupy the binding pockets of the different EH domains. We provide a detailed molecular characterization of these intra-molecular interactions within Eps15, which are likely key to the liquid-liquid phase separation of Eps15 in the absence of other endocytic proteins. Our NMR results indicate that both Dab2_320–495_ and Eps15_IDR_ can bind EH123 simultaneously, but partially compete with each other. In line with this, we observe that Dab2_320–495_ is recruited into liquid-like condensates formed by full-length Eps15. Our results provide a detailed description of the multivalent and promiscuous interactions between Eps15 and Dab2, which appear to be key to a dynamic protein network that can phase separate and evolve over time, which is required in the early stages of CME.

## Results

### Dab2_320–495_ is an IDR with mild helical propensities

The around 500 residue long IDR of Dab2 comprises a central region, which is predicted by IUPred2A^23^ to be significantly more disordered than the remainder of the IDR (Fig. 1B). We designed a construct encompassing this region, reaching from residue 320 to 495 (Dab2_320–495_). This region contains 2 putative binding sites for clathrin (DLL/DLF) and one NPF motif, prone to bind to EH domains contained in Eps15. In order to assess this construct by NMR spectroscopy and describe its conformational sampling, which is fundamental to its function, we first expressed and purified the construct, and assigned the NMR backbone resonances of a ^1^H, ^15^N, ^13^C labeled sample (Supplementary Fig. 1). We were initially not able to assign two small regions within Dab2_320–495_, for which we designed two new stretches (Dab2_328–360_ and Dab2_358–390_), whose ^1^H-^15^N heteronuclear single quantum coherence (HSQC) spectrum overlaid well with the one of Dab2_320–495_ (Supplementary Fig. 2), enabling the transfer of assignments from Dab2_328–360_ and Dab2_358–390_ to Dab2_320–495._ As predicted, Dab2_320–495_ appears to be disordered along its entire length, testified by secondary chemical shifts (SCSs) close to zero (Supplementary Fig. 3). However, calculating a conformational ensemble on the basis of chemical shifts using a combination of the statistical coil generator flexible meccano^24^ and the selection algorithm ASTEROIDS^25^ revealed three regions with mild helical propensity as compared to random coil: 339–354, 454–460, and 486–490 (Supplementary Fig. 3). Around the NPF motif, no particular structural propensity could be observed. We then recorded ^15^N spin relaxation rates, exquisitely sensitive to the fast motions that intrinsically disordered proteins (IDPs) and IDRs undergo, in order to understand the dynamics of the intrinsically disordered chain of Dab2_320–495_. In agreement with the calculated SCSs, ^15^N spin relaxation rates (R_1ρ_, R_1_, {^1^H}-^15^N heteronuclear Overhauser effect - hetNOE) are reminiscent of those of an intrinsically disordered protein (Supplementary Fig. 4), with increased rigidity, testified by increased R_1ρ_ and hetNOE rates, apparent around the transient helix between residues 328 and 360 (helix_N_). This helix is connected with the remainder of the disordered chain by a few amino acids of more rapid mobility. The more C-terminal transient helices are also characterized by slightly increased R_1ρ_ and hetNOE rates, although less pronounced than those of helix_N_.

### EH2 and EH3 are promiscuous binders

**Fig. 2:**
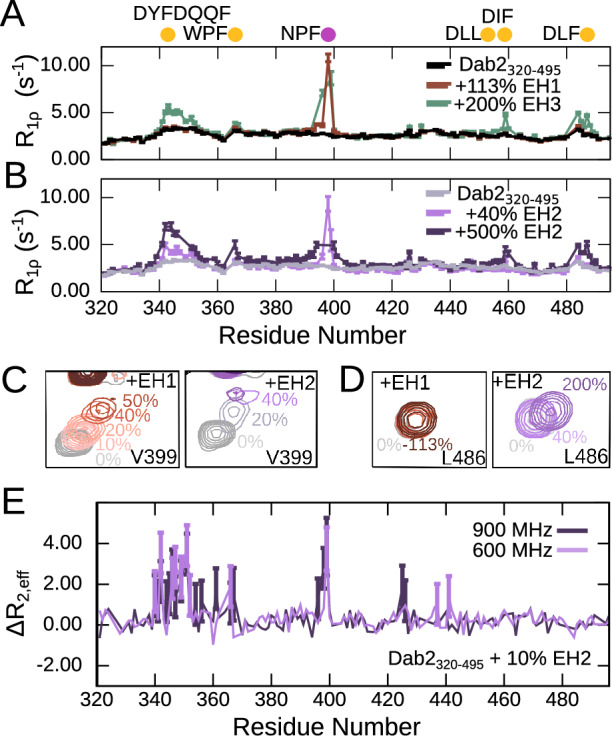
^15^N spin relaxation of Dab2_320–495_ upon interaction with the EH domains. ^15^N R_1ρ_ spin relaxation of Dab2_320–495_ (100 µM) in the absence and presence of **(A)** EH1 or EH3 and **(B)** increasing concentrations of EH2. The experiments were recorded at a ^1^H frequency of 600 MHz. The relaxation rates in (**A**) and (**B**) were derived from a fit of peak intensities against the relaxation delay. Errors of the fitted rates were derived from the experimental uncertainty. **C** Zoom into the peak corresponding to V399 from Dab2_320–495_^1^H-^15^N HSQC spectra alone and in the presence of increasing concentrations of EH1 (left) and EH2 (right). **D** Zoom into the peak corresponding to L486 from Dab2_320–495_^1^H-^15^N HSQC spectra alone and in the presence of increasing concentrations of EH1 (left) and EH2 (right). Color legends are displayed in the respective panels. **E** ΔR_2, eff_ calculated from effective R_2_ values (R_2, eff_) of relaxation dispersion experiments at a CPMG frequency of 31 Hz subtracted with R_2, eff_ at a CPMG frequency of 1000 Hz recorded on a sample of 100 µM Dab2_320–495_ in the presence of 10% EH2 at a ^1^H frequency of 600 MHz and 900 MHz. Error bars, propagated from the errors of R_2, eff_ at 31 Hz and 1000 Hz determined from repeat measurements and with a minimum error of 0.5 (see Supplementary Fig. 7B for the corresponding decay curves), are shown for values that are significantly larger than 0 .

We then titrated the different Eps15 EH domains (EH1, EH2, or EH3) into ^15^N Dab2_320–495_ and recorded ^1^H-^15^N HSQC spectra at the different titration steps to assess which residues in Dab2 take part in the interaction. While small chemical shift perturbations (CSPs) are observed very locally upon addition of EH1, the spectrum of Dab2_320–495_ is more significantly perturbed when EH2 and EH3 are present at comparable concentrations (Fig. 1C–F and Supplementary Figs. 5, 6). Plotting the CSPs of Dab2_320–495_ upon interaction with the different EH domains along its sequence (Fig. 1D–F) clearly illustrates that EH1, EH2, and EH3 have different binding patterns: EH1 is the most selective of all Eps15 EH domains, binding mainly to the NPF motif of ^15^N Dab2_320–495,_ while very small CSPs occur around residue F366 only at 200% EH1. EH2 and EH3, on the other hand, were able to interact with multiple small stretches on Dab2_320–495_, revealing a high degree of binding promiscuity. This binding behavior is also mirrored by decreased peak intensities (Supplementary Fig. 6) and increased R_1ρ_ rates around the respective interaction sites (Fig. 2A, B). Since a spin-lock field of 1500 Hz was used in the R_1ρ_ experiments, effectively quenching contributions of intermediate exchange to the relaxation rates, the observed increases in R_1ρ_ reflect the slowed rotational tumbling times of the interacting residues when in contact with the respective EH domain. Residues in between the interaction sites have R_1ρ_ rates similar to those of the unbound Dab2_320–495_, suggesting that the different interaction sites act independently from each other. The acquisition of R_1ρ_ experiments of the different titration steps thus provides critical information on the dynamic behavior of Dab2’s intrinsically disordered chain and how this is affected by binding to the folded – and much more slowly tumbling – EH domains. Even though the largest CSPs were observed around the NPF motif when EH2 and EH3 were added, also residues around DYF, WPF, DLF, and DIF motifs were in contact with those two EH domains at higher EH:Dab2 ratios. This is also reflected by the ^15^N R_1ρ_ rates measured (Fig. 2A, B). Common to all motifs is the phenylalanine residue, suggesting an importance for this residue type in the binding to EH2 and EH3 (Fig. 1E, F). Interestingly, most of these motifs are located in the regions with increased helical propensity of Dab2_320–495_ (Supplementary Fig. 3).

The Dab2_320–495_ motifs were furthermore observed to bind to the EH domains with different dynamics. For example, V399, just next to the NPF motif, showed only mild peak broadening upon interaction with EH1, justified by the slowed rotational tumbling times when in complex. Upon interaction with EH2, however, significantly stronger broadening was observed (Fig. 2C). The other interacting regions show very little broadening and are mainly characterized by CSPs. One example is the residue L486, which does not interact with EH1 and shows essentially no line broadening combined with small CSPs in the presence of EH2 (Fig. 2D). These differential behaviors prompted us to record Carr-Purcell-Meiboom-Gill (CPMG) relaxation dispersion of Dab2_320–495_ to investigate the possibility of intermediate exchange in the microsecond to millisecond regime between the bound and unbound states in the presence of EH2 (Fig. 2E). These experiments confirm that the NPF motif binds EH2 in intermediate exchange on the NMR chemical shift timescale. Surprisingly, many residues throughout the helix_N_ also displayed dynamics on the intermediate timescale upon binding. The remaining motifs, DIF and DLF, were concluded to bind in fast exchange.

In order to quantify the affinity of the interaction between Dab2_320–495_ and the EH domains, we attempted to extract dissociation constants (K_D_) for the different Dab2_320–495_ interaction motifs. Even though fitting of CSPs as a function of EH domain concentration was difficult due to the low affinities and peak broadening (especially around the NPF motif upon interaction with EH2), they can be estimated to be in the high micromolar to millimolar range. The highest affinity of ~340 μM was determined between residue G400 and EH2—this residue is close to the NPF motif, which broadens too rapidly in the titration to determine a K_D_. EH1 interacts with the NPF motif (G400) with an affinity similar to that of EH2 (~350 μM), while the affinity of EH3 was estimated to only ~1.4 mM. The other, promiscuous binding sites, interact even more weakly (Supplementary Fig. 7A and Supplementary Table 1). Analysis of the CPMG relaxation dispersion curves of Dab2_320–495_ upon interaction with EH2 using a two-site exchange model allowed us to extract an exchange rate of 149 ± 13 s^−1^ and a percentage of bound Dab2_320–495_ of 3.3 ± 0.3% for protein concentrations of 100 μM Dab2_320–495_ and 10 μM EH2 (Supplementary Table 2). This results in a K_D_ value of 196 μM, which is in a similar range as the affinity between Dab2_320–495_ and EH2 determined by CSPs. The dispersion in the helix_N_ and around the NPF motif were fit together in the fits of the CPMG experiments (Supplementary Fig. 7B).

### Dab2_320–495_ binds to the hydrophobic pocket of EH domains

**Fig. 3:**
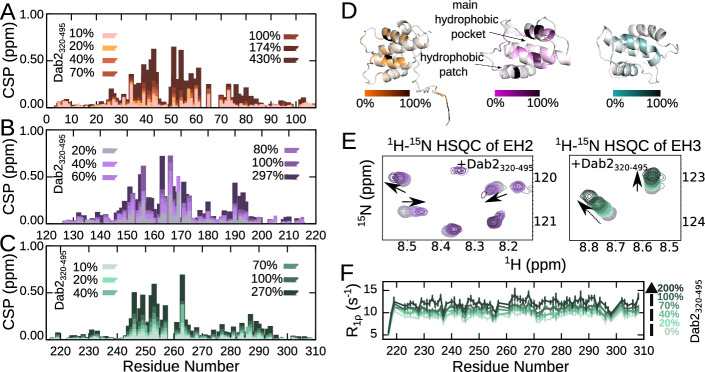
Interaction between the individual EH domains of Eps15 and Dab2_320–495_. **A–C** CSPs of 100 μM EH1 (**A**), EH2 (**B**) and EH3 (**C**), respectively, in the absence versus the presence of increasing amounts of Dab2_320–495_. Color legends are displayed in the respective plots. **D** Structures of EH1 (AlphaFold2^34^), EH2 (PDB 1FF1^31^) and EH3 (PDB 1C07^32^) with the CSP at 100% Dab2_320–495_ mapped onto them. The linear gradient starts from 0% (white), which represents no CSP, to the 100% (black), which is 0.75 ppm (the biggest CSP observed between EH2 and Dab2_320–495_). **E** Zoom into a ^1^H-^15^N TROSY-HSQC spectrum of EH2 (left) and EH3 (right) alone and in the presence of Dab2_320–495_. **F **^15^N R_1ρ_ spin relaxation of EH3 (100 µM) in the absence and presence of Dab2_320–495_ at a ^1^H frequency of 600 MHz. Color legends are indicated in the figures. The relaxation rates were derived from a fit of peak intensities against the relaxation delay. Errors of the fitted rates were derived from the experimental uncertainty.

We wondered whether differences in the EH domains could explain the distinct interactions observed with the different motifs of Dab2_320–495_. We thus set out to determine the binding pockets on each of the EH domains that engage in interaction with Dab2_320–495_. Even though NMR assignments of the three EH domains exist^26–28^, we assigned all three EH domains under our experimental conditions (Supplementary Figs. 8–10). From the carbon SCSs, we calculated secondary structure propensities (SSPs)^29^, which are in agreement with previously determined NMR structures of the proteins (Supplementary Fig. 11)^30–32^. Additionally, ^15^N R_1_ and R_1ρ_ relaxation rates reflect small folded domains with flexible N- and C-termini, as expected (Supplementary Fig. 12). We then titrated Dab2_320–495_ into the different ^15^N labeled EH domains and recorded ^1^H-^15^N Transverse relaxation-optimized spectroscopy (TROSY) HSQC spectra of the EH domains at the different titration steps. We plotted the respective CSPs along the sequence of the individual EH domains (Fig. 3A–C) and then colored the structures of the three EH domains relative to the magnitude of the observed CSPs (Fig. 3D). Zooms into the spectra of EH2 and EH3 illustrate the spectral changes upon Dab2_320–495_ binding (Fig. 3E). The largest CSPs occur around the previously identified binding pockets^18,31^ and are comparable between all three EH domains (Fig. 3D). An additional binding region, a hydrophobic patch previously shown to bind a second NPF motif in Stonin2^18^, is clearly visible in EH2. The region seems to participate in binding also within EH1 and EH3, albeit in a less pronounced way (Fig. 3A-D, region around residues 185–195 in EH2). R_1ρ_ relaxation rates systematically increase throughout all residues when Dab2_320–495_ is titrated into the ^15^N EH domains, likely reflecting mildly increased tumbling times resulting from the motional drag that the bound IDR exerts (Fig. 3F)^33^.

### EH123 shows increased binding promiscuity

**Fig. 4:**
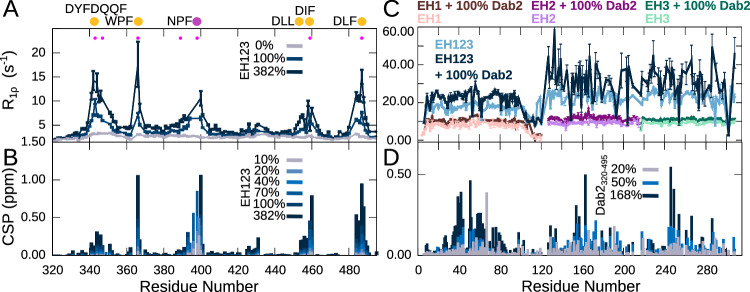
Interaction between EH123 and Dab2_320–495_. **A**^ 15^N R_1ρ_ spin relaxation of Dab2_320–495_ (100 µM) in the absence and presence of increasing concentrations of EH123 at a ^1^H frequency of 600 MHz. **B** CSPs between the ^1^H-^15^N HSQC of ^15^N Dab2_320–495_ in the absence and presence of increasing amounts of EH123. The filled pink dots represent the phenylalanines present in the Dab2_320–495_. **C**^ 15^N R_1ρ_ spin relaxation of the individual ^15^N EH domains (EH1, EH2, EH3) as well as EH123 in the absence and presence of 100% Dab2_320–495_ at a ^1^H frequency of 600 MHz. **D** CSPs between the ^1^H-^15^N TROSY-HSQC of ^15^N EH123 in the absence and presence of increasing amounts of the Dab2_320–495_. The color legends are indicated in the respective figure panels. The relaxation rates in (**A**) and (**C**) were derived from a fit of peak intensities against the relaxation delay. Errors of the fitted rates were derived from the experimental uncertainty.

In order to better reflect the physiological scenario, where the different EH domains in Eps15 are not isolated, but expressed in row, we investigated the interaction between Dab2_320–495_ and the full EH-domain containing region (EH1-EH2-EH3, also called EH123) by titrating increasing concentrations of EH123 into ^15^N Dab2_320–495_. As expected from the interactions of the individual EH domains, both CSPs and R_1ρ_ rates reveal that EH123 is able to interact with the same regions of Dab2_320–495_ as observed for EH2 and EH3 (Fig. 4A, B). Interestingly, the R_1ρ_ relaxation rates of Dab2_320–495_ in the presence of EH123 show increased relaxation not only of the residues of the five Dab2_320–495_ binding regions, but the residues located in between the binding motifs also show mildly increased rates. This is particularly evident for the residues located between the first three binding motifs (DYFDQQF, WPF and NPF) and suggests an overall stiffening of the chain, potentially due to binding of the motifs to different EH domains within the same EH123 molecule.

While CSPs around the NPF motif remain the largest upon interaction with EH123, the difference between the interaction of EH123 with the NPF motif and the other motifs appears smaller than for interaction with the individual EH domains. Indeed, R_1ρ_ rates increase much more significantly around these promiscuous sites, albeit part of the increase should be attributed to the larger size of EH123 relative to the individual domains. Nonetheless, binding affinities approximated from the CSPs for EH123 binding are significantly higher than those of the individual EH domains, ranging from around 100 to around 400 μM (Supplementary Fig. 7A and Supplementary Table 1). This suggests that once one EH domain within EH123 is bound to Dab2_320–495_, the two other EH domains of the same molecule remain available for (preferential) binding to other motifs, an effect reminiscent of avidity, effectively increasing the total level of binding promiscuity. A much higher concentration of individual EH domains (3x as many) is needed to partially recover the chemical shift differences and R_1ρ_ rates of Dab2_320–495_ in the presence of EH123 (Supplementary Fig. 13).

### All EH domains within EH123 engage in interaction with Dab2

To investigate whether Dab2_320–495_ binds to the same sites on the EH domains when they are expressed in row rather than independently, we also aimed to characterize the interaction between Dab2_320–495_ and EH123 from the side of EH123. We therefore produced ^15^N labeled EH123 and recorded a ^1^H–^15^N TROSY-HSQC spectrum. If the different EH domains were unaffected by being connected to each other, we would expect this spectrum to overlay perfectly with those of the individual EH domains. However, small CSPs were observed for all three EH domains, and the peaks of EH2 and EH3 appeared broadened (Supplementary Fig. 14), revealing that each EH domain is affected by the presence of the two additional domains, potentially due to intra-molecular interactions. Despite the changes in the spectrum of EH123 compared to those of EH1, EH2, and EH3, the assignments from the individual domains could be fully transferred to the combined construct. In order to examine the broadening of the peaks originating from EH2 and EH3, we recorded ^15^N spin relaxation of EH123 (R_1ρ_). All R_1ρ_ rates of EH123 were significantly increased as compared to the individual EH domains, which was expected for this larger protein construct (Fig. 4C and Supplementary Fig. 14C). The rates of EH2 and EH3, however, were significantly higher overall than those from EH1. Indeed, the increased R_1ρ_ rates were similar for EH2 and EH3, suggesting that EH2 and EH3 tumble together as one entity, while EH1 can move independently within EH123. To further investigate this behavior, we decided to address the very fast, picosecond motions that are normally present in dynamic linkers between folded domains by measuring a hetNOE experiment with EH123. The recorded hetNOEs show quite similar values all along EH123, reflecting the similar fold and internal dynamics of the individual domains (Supplementary Fig. 14C). Only the linker between EH1 and EH2 displays much lower hetNOE values, demonstrating that this linker is undergoing much more rapid motion than the rest of the protein construct, also compared to the linker connecting EH2 and EH3. The hetNOE data are thus in very good agreement with EH2 and EH3 tumbling together within the EH123 construct.

We then added Dab2_320–495_ to ^15^N labeled EH123 to assess whether EH123 bound differently to Dab2 than the individual EH domains. We observed CSPs for all three domains, similar to those of the individual EH domains when interacting with Dab2_320–495_ (Fig. 4D), suggesting that the binding sites on all EH domains are also available within EH123, despite the fact that EH2 and EH3 tumble together within EH123. R_1ρ_ rates increased throughout EH123 when Dab2_320–495_ was added (Fig. 4C), and the rates of EH1 remained lower than those of EH2 and EH3, suggesting that the overall conformation of the EH domains within EH123 is preserved upon binding of Dab2_320–495_.

### Eps15’s own IDR interacts with EH123

**Fig. 5:**
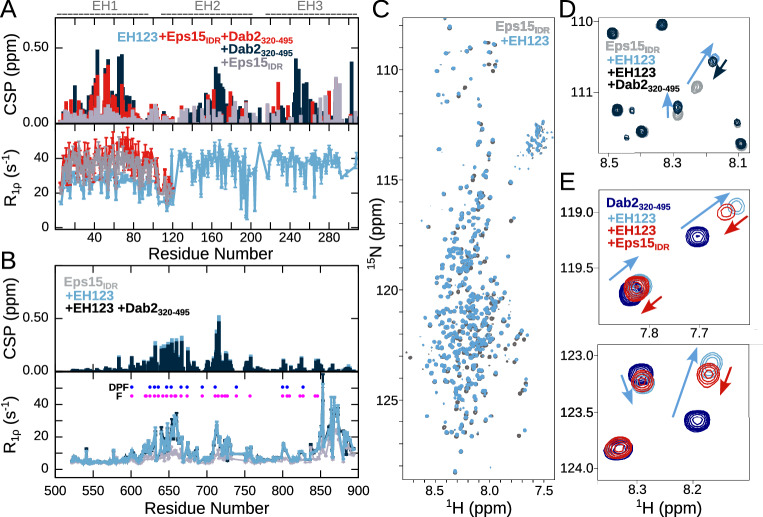
Competitive binding of Dab2_320–495_ and Eps15_IDR_ to EH123. **A** CSPs calculated between the ^1^H–^15^N TROSY-HSQC of 200 µM ^15^N EH123 in the absence and presence of 200 µM Dab2_320–495_ and/or 200 µM Eps15_IDR._ Below are ^15^N R_1ρ_ spin relaxation rates of ^15^N EH123 in the absence and presence of 100% Eps15_IDR_ and both 100% Dab2_320–495_ and Eps15_IDR_ at a ^1^H frequency of 1200 MHz. Only the R_1ρ_ rates within EH1 in the presence of Eps15_IDR_ and Eps15_IDR_ with Dab2_320–495_ are shown as the peaks of EH2 and EH3 were severely broadened. The parts of EH123 corresponding to EH1, EH2, and EH3 are illustrated above the plots. **B** CSPs calculated between the ^1^H-^15^N HSQC of 100 µM ^15^N Eps15_IDR_ in the absence and presence of 100 µM EH123 and 100 µM EH123 + 100 µM Dab2_320–495_, and ^15^N R_1ρ_ spin relaxation of ^15^N Eps15_IDR_ in the absence and presence of 100% EH123 and 100% EH123 + 100% Dab2_320–495_ at a ^1^H frequency of 1200 MHz. **C** ^1^H–^15^N HSQC spectra of 100 µM ^15^N Eps15_IDR_ alone and in the presence of 100 µM EH123. **D** Zoom into ^1^H-^15^N HSQC spectrum of 100 µM ^15^N Eps15_IDR_ alone and in the presence of 100 µM EH123 and 100 µM EH123 + 100 µM Dab2_320–495_. **E** Zoom into ^1^H-^15^N HSQC spectrum of 100 µM ^15^N Dab2_320–495_ alone and in the presence of 100 µM EH123 and 100 µM EH123 + 100 µM Eps15_IDR_. Color codes are denoted in the respective panels. The relaxation rates in (**A**) and (**B**) (lower panels) were derived from a fit of peak intensities against the relaxation delay. Errors of the fitted rates were derived from the experimental uncertainty.

The remarkable binding promiscuity towards phenylalanine-containing motifs observed between EH123 and Dab2_320–495_, prompted us to investigate whether EH123 could also interact with Eps15’s own IDR (Eps15_IDR_). Eps15_IDR_ does not contain any canonical NPF motifs, but contains 14 DPF motifs (Fig. 1A), which have previously been suggested to interact with Eps15’s own EH domains^16^. When we added equimolar amounts of Eps15_IDR_ to ^15^N EH123, CSPs were observed throughout the ^1^H–^15^N TROSY-HSQC spectrum of EH123, confirming the interaction and further suggesting that all EH domains bind to Eps15_IDR_ (Fig. 5A and Supplementary Fig. 15). Upon interaction, most peaks of EH2 and EH3 were severely broadened, likely because of the motional drag the long Eps15_IDR_ exerts on EH123. As a consequence, many of the signals were too weak to calculate CSPs and extract relaxation rates for EH2 and EH3 within EH123 upon interaction with Eps15_IDR_ (Fig. 5A). However, the CSPs originating from EH1 within EH123 were all smaller than those in presence of the same amount of Dab2_320–495_, indicating that EH1 interacts less well with Eps15_IDR_ than with Dab2_320–495_, in agreement with EH1 being the least promiscuous of the domains, binding more selectively to NPF motifs than EH2 and EH3 (Fig. 5A).

### EH123 binds phenylalanine-containing motifs in Eps15_IDR_

In order to further characterize the interaction between Eps15_IDR_ and EH123, we assigned the backbone resonances of ^1^H, ^15^N and ^13^C labeled Eps15_IDR_. To circumvent the spectral overlap of the long Eps15_IDR_, we designed four overlapping smaller stretches (Eps15_IDR 481-581_, Eps15_IDR 569–671_, Eps15_IDR 648-780_, and Eps15_IDR 761–896_). The ^1^H-^15^N HSQC spectra of these stretches overlayed nicely with the spectrum of the full Eps15_IDR_ (Supplementary Fig. 16), allowing the transfer of ^1^H and ^15^N resonance assignments between the spectra. We assigned 242 out of 393 resonances distributed across the IDR except for the N-terminal residues 481-521 to which no resonances could be assigned (Supplementary Fig. 17). This is likely because these residues (481–504) are part of the coiled-coil domain as predicted by AlphaFold2^34^, giving rise to weak peaks in the 3D assignment spectra. Low R_1_ and R_1ρ_ relaxation rates and SCSs around 0 for both Eps15_IDR_ and the smaller stretches agree well with a disordered protein (Supplementary Fig. 18). The C-terminal part however, (~residues 850–885) has increased R_1ρ_ rates and SSP values close to 1, revealing a stable alpha helix. This alpha helix, which is also predicted by AlphaFold2^34^, contains the two ubiquitin interaction motifs (UIMs) present in Eps15^35,36^. In general, the R_1ρ_ relaxation rates are slightly larger within Eps15_IDR_ compared to the smaller stretches, potentially resulting from small hydrophobic clusters in these regions leading to transient self-interactions within the chain—an effect that has been observed for transiently folded elements in other IDPs^37^.

We next recorded ^1^H-^15^N HSQC spectra of ^15^N Eps15_IDR_ with equimolar amounts of unlabeled EH123 in order to pinpoint the exact interaction regions on Eps15_IDR_. R_1ρ_ rates, CSPs, and intensity ratios compared to Eps15_IDR_ on its own revealed two relatively large interaction regions (Fig. 5B, C and Supplementary Fig. 19). These regions, 620–680 and 700–730, are characterized by a high density of phenylalanines. While the first region is enriched in DPF motifs, which have previously been proposed to bind to EH123, the second region is characterized by fewer DPF motifs and contains multiple other small linear motifs with phenylalanine, underlining the promiscuity of the EH domains. Moreover, small CSPs and increases in R_1ρ_ rates are observed around three smaller regions, indicating additional interaction sites for EH123: residues ~800–809 and ~820–827 containing DPF(s), and residues ~843–846 containing phenylalanines. While increases in R_1ρ_ relaxation rates and decreases in intensity ratios in the C-terminal helix are also observed (Fig. 5B and Supplementary Fig. 19), the small CSPs accompanying these changes suggest that this may be through binding to the first helical residues of hydrophobic nature (843–846), which then affects the tumbling time of the entire alpha helical element.

### Dab2_320–495_ and Eps15_IDR_ partially compete for EH123 binding

The interaction observed between EH123 and Eps15_IDR_ suggests that intra-molecular interactions within Eps15 may occupy Dab2 binding sites on the EH domains in the native context. We therefore conducted a competition experiment, acquiring a spectrum of ^15^N EH123 with equimolar amounts of both Eps15_IDR_ and Dab2_320–495_. The resulting CSPs of EH123 showed that in the presence of both Dab2_320–495_ and Eps15_IDR_, the CSPs of EH1 were similar to those in the presence of Dab2 only. R_1ρ_ rates around EH1 were increased mildly as compared to binding of Eps15_IDR_ or Dab2_320–495_ alone to EH123, while the peaks of EH2 and EH3 were broadened severely, also as observed upon Eps15_IDR_ interaction (Fig. 5A and see Supplementary Fig. 15B). While it is difficult to disentangle the individual contributions of Dab2_320–495_ and Eps15_IDR_ binding to the spectral changes, this suggest that both IDRs may bind EH123 at the same time. To further investigate this, we recorded a ^1^H-^15^N HSQC spectrum of ^15^N Eps15_IDR_ and added first EH123 and then Dab2_320–495_. While most peaks were unaffected by the addition of Dab2_320–495_, some peaks shifted very slightly back towards the unbound state of Eps15_IDR_ (Fig. 5B, D and Supplementary Fig. 19). This suggests that binding between Eps15_IDR_ and EH123 is largely unaffected by the presence of Dab2_320–495_. We then conducted the same competition experiment, but this time using ^15^N Dab2_320–495_. Again, when adding equimolar amounts of Eps15_IDR_ to ^15^N Dab2_320–495_ bound to EH123, the spectrum of Dab2_320–495_ was only slightly affected, with peaks from bound Dab2_320–495_ moving towards the unbound form (Fig. 5E and Supplementary Fig. 20), likely due to competition with Eps15_IDR_. To rule out that any of the observed spectral changes could originate from a potential interaction between Dab2_320–495_ and Eps15_IDR_, we recorded a ^1^H-^15^N HSQC spectrum of ^15^N Dab2_320–495_ with Eps15_IDR_, confirming that the two IDRs do not interact (Supplementary Fig. 21). Taken together, the competition experiments conducted from the side of each binding partner (EH123, Eps15_IDR_, and Dab2_320–495_) suggest that both Eps15_IDR_ and Dab2_320–495_ can bind to EH123 at the same time, even though they are competing for the same EH domain interaction sites. This could be possible due to a fast on and off rate of the low-affinity interaction motifs, provided binding sites in both Dab2_320–495_ and Eps15_IDR_ bind with similar affinities, thereby creating a complex and dynamic interaction network allowing both IDRs to bind EH123.

### Dab2_320–495_ enters into Eps15 protein condensates

**Fig. 6:**
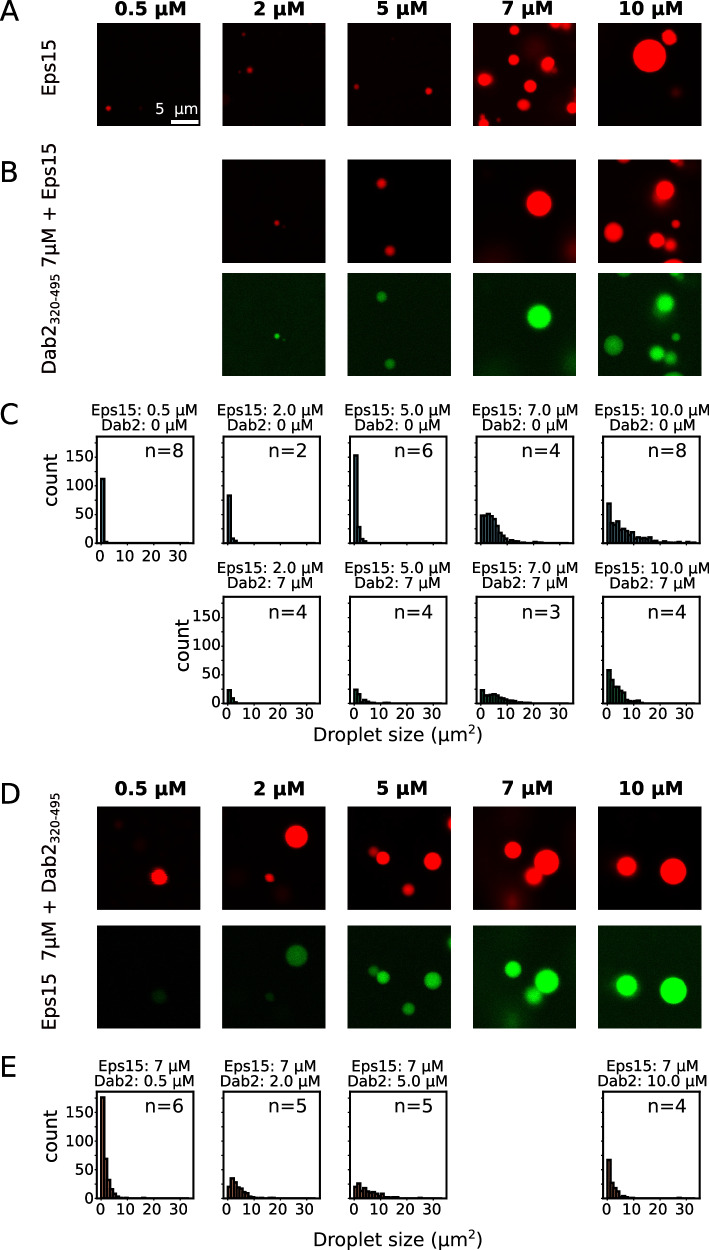
Dab2_320–495_ is recruited into Eps15 droplets. **A** Representative droplets imaged from Eps15 alone at different concentrations (0.5 to 10 μM, indicated above). **B** Eps15 droplets formed under the same conditions in the presence of 7 μM Dab2_320–495_. **C** Droplet sizes plotted in a histogram for different concentrations of Eps15 in the presence and absence of 7 μM Dab2_320–495_, corresponding to the microscopy images in (**A**) and (**B**). **D** Eps15 droplets formed with 7 μM protein, to which different concentrations of Dab2_320–495_ (0.5 to 10 μM, indicated above) was added. **E** Droplet sizes plotted in a histogram for different concentrations of Dab2_320–495_ in the presence and absence of 7 μM Eps15, corresponding to the microscopy images in (**D**). 10% of the protein used in the microscopy experiments was fluorescently labeled. Eps15 was labeled using AZDye594 (displayed in red). Dab2_320–495 S328C_ was labeled using AZDye488 (displayed in green). The scale bar is 5 μm and applies to all images in the figure. Images were acquired by confocal microscopy. The number of images used for the histograms is indicated as ‘n = x’ in each plot. Source data are the same as for Supplementary Fig. 22A and B and are provided as a Source Data file.

Eps15 has recently been shown to form liquid-like droplets, and interactions between the EH domains and Eps15_IDR_ were proposed to contribute to droplet formation^16^. The seemingly simultaneous interactions of Eps15_IDR_ and Dab2_320–495_ with EH123 observed here made us question whether Dab2_320–495_ could be recruited to Eps15 condensates. We therefore expressed and purified full-length Eps15, from here on just called Eps15, and labeled its cysteines with the fluorophore AZDye594. In order to fluorescently label Dab2_320–495_, which does not contain any cysteines, we created a single cysteine mutant (S328C Dab2_320–495_), and labeled it with AZDye488. We first examined if we could achieve liquid-like droplet formation of Eps15 under our experimental conditions. Indeed, upon addition of 3% PEG8000, we observed droplet formation starting from a protein concentration of as little as 0.5 μM (Fig. 6A). Droplets increased in size with increasing Eps15 concentrations (Fig. 6A, C and Supplementary Fig. 22A). Unlike Eps15, Dab2_320–495_ was not able to form any droplets on its own (Supplementary Fig. 23B). When Dab2_320–495_ was added to Eps15 droplets, it entered readily and clearly enriched in the Eps15 condensed phase (Fig. 6B). While the presence of Dab2_320–495_ did not majorly alter the size or number of the droplets overall, we did not manage to observe phase separation at the lowest tested concentration of 0.5 μM Eps15 when 7 μM Dab2_320–495_ was present in addition (Fig. 6B, C and Supplementary Fig. 22). We were thus wondering, how Dab2_320–495_ concentration affected Eps15 condensation. Therefore, we added increasing concentrations of Dab2_320–495_ (0.5 to 10 μM) to droplets formed by 7 μM Eps15—a concentration at which many and relatively large droplets can be observed. Dab2_320–495_ entered into the droplets at all concentrations added, with the intensity of Dab2_320–495_ inside the droplets increasing with higher Dab2_320–495_ concentration (Fig. 6D, Supplementary Fig. 22). No major effects on condensate size or number of condensates were observed (Fig. 6E and Supplementary Fig. 22), and even 20 times molar excess of Dab2_320–495_ did not seem to majorly alter the condensates (Supplementary Fig. 23). Since droplets made in the presence of smaller concentrations of Dab2_320–495_ were significantly less bright than those made in the presence of higher Dab2_320–495_ concentrations, we also analyzed the partition coefficient of both proteins into the droplets. While Eps15 partitioned better into droplets with increasing Eps15 concentration, its partitioning was less dependent on Dab2_320–495_ concentration (Supplementary Fig. 22C, D). Partitioning of Dab2_320–495_ into the droplets depended on both its own concentration in the sample, as well as the concentration of Eps15 (Supplementary Figs. 22C, D). Even though the interactions between Dab2_320–495_ and EH123 are rather weak, recruitment into Eps15 droplets is specific, illustrated by a fluorescently labeled DNA, which is not recruited into Eps15 droplets (Supplementary Fig. 23E). This indicates, in line with our NMR results, that the inter-molecular Eps15 interactions responsible for droplet formation can be maintained in the presence of Dab2_320–495_, while Eps15 also remains available for interaction with Dab2.

## Discussion

The dynamic network formed by intrinsically disordered regions (IDRs) and folded proteins interacting with short linear motifs within these IDRs, is key to a number of biological processes^4,38^. Clathrin-mediated endocytosis is a prime example of a process that comprises many of those short linear motifs^2,21^, leading to the formation of a complex interaction network. Known examples are the α and β2 appendage domains of the major adapter protein AP2 that interact with DPF/DPW and FxDxF motifs (x = any amino acid)^3^, the clathrin heavy chain terminal domain that interacts with DLL/DLF motifs^7,8^ or the EH domains found in Eps15, which interact with NPF motifs of diverse IDR partners^18^. Although extensive studies have identified these consensus sequences, it has meanwhile become clear that additional binding sites are yet to be discovered and that this requires a strategy by which focus lies on studying long endocytic IDRs. This has recently allowed identifying a particularly long interaction site between the neuronal AP180 and the AP2β2 appendage domain^7^. In addition, many endocytic IDRs seem to bind to the same partners^39^ and understanding how the different binding modes synergize or compete with each other is crucial for a molecular comprehension of clathrin-mediated endocytosis.

**Fig. 7:**
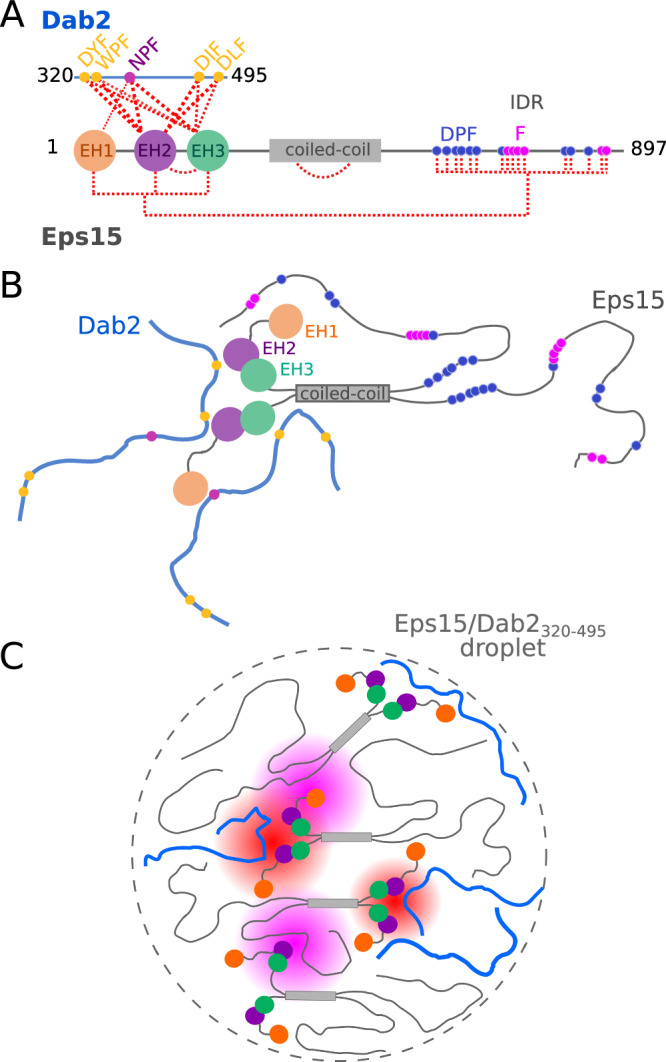
Schematic of Eps15 and Dab2_320–495_ interactions and phase separation. **A** illustration of the complex interaction network between Eps15 and Dab2_320–495_, including intra-molecular interactions within Eps15. Interactions are visualized with red dashed lines. **B** Cartoon of self-interactions within Eps15 and of interactions between Eps15 and Dab2_320–495_. Eps15’s EH domains interact with Eps15_IDR_ and with Dab2_320–495_. EH2 and EH3 tumble together. All three EH domains interact with the NPF motif within Dab2_320–495_, while EH2 and EH3 are promiscuous binders. **C** The weak multivalent interaction network of the two IDRs with EH123 likely contributes to co-phase separation of Eps15 and Dab2_320–495_. Eps15:Dab2 interactions are highlighted in red shading, and Eps15:Eps15 interactions are highlighted in pink shading.

In this study, we shed light onto those partially competitive interactions by investigating the binding of a large intrinsically disordered region stemming from the CLASP Dab2 (Dab2_320–495_) and its interactions with Eps15 EH domains, as well as its competition for binding with Eps15’s own IDR (Eps15_IDR_). In line with the previous literature and extensive peptide screens to assess binding patterns of Eps15 EH domains^40^, all three EH domains of Eps15 (EH1, EH2 and EH3) prefer to bind to the only NPF motif within the sequence of Dab2_320–495_ (Fig. 1, 2). In the presence of EH2, significant exchange in the microsecond to millisecond time scale is observed around the NPF motif and an N-terminal transient helix in Dab2_320–495_ (helix_N_), allowing to estimate a dissociation constant between Dab2_320–495_ (NPF motif and helix_N_) and EH2 on the order of hundreds of micromolar (Fig. 2, Supplementary Fig. 7B and Supplementary Table 2). These affinities are in agreement with affinities of other small linear motif interactions determined in the context of clathrin-mediated endocytosis^7–9^ and also with those determined from chemical shift perturbations between Dab2_320–495_ and EH domains (Supplementary Fig. 7A and Supplementary Table 1). However, they are orders of magnitude weaker than those observed between Stonin2 and EH2 (K_D_ in the nanomolar range)^18^. Although the EH domains bind preferentially to the NPF motif of Dab2_320–495_, as it is expected from the literature^18,31,32,40^, the atomic resolution provided by our NMR experiments also points toward additional, slightly weaker binding sites including DYF, WPF, DLF, and DIF motifs (Fig. 1). Such sites will certainly play a role in the crowded environment of the endocytic pit, and these motifs further testify to an extremely promiscuous binding behavior of EH2 and EH3. This is in line with the observation that other EH domains, such as the EH domain of EHD1 or of POB-1 can bind to the motif xPF^41,42^. By investigating the EH domain interactions with small linear motifs from the side of a bona fide IDR, we can conclude that the promiscuity of EH2 and EH3 is even larger than previously anticipated, binding to most phenylalanine containing motifs (Fig. 7A). It is remarkable that, overall, the dissociation constants determined for binding between EH domains and the NPF motif in Dab2_320–495_ are not extremely different to those between EH domains and non-NPF binding sites (~1.5 mM versus up to ~4 mM for EH3 for example, ~0.3 mM versus up to ~10 mM for EH2), suggesting that promiscuity can indeed play an important role physiologically.

Even though some proteins contain individual EH domains, this is not the case for Eps15, which contains EH1, EH2 and EH3 sequentially at its N-terminus. Unexpectedly, we observe that EH2 and EH3 move together as one entity in a construct comprising all three EH domains (EH123, Fig. 4C), suggesting a potential interaction between EH2 and EH3. EH1, on the other hand, tumbles independently from the other two EH domains. Nonetheless, EH123 makes all EH domain binding sites available for interaction with Dab2_320–495_. From the side of Dab2_320–495_, binding to EH123 is strikingly different than binding to the individual EH domains. While the same NPF and promiscuous motif interactions maintain, the non-NPF interactions seem to gain importance, leading to affinities between the different Dab2_320–495_ motifs in the hundreds of μM range for both NPF and non-NPF motifs (Fig. 4, Supplementary Figs. 7A, 13 and Supplementary Table 1). This could be due to avidity effects of the three EH domains in close spatial proximity compared to the individual domains.

The present promiscuity of interactions, particularly in the context of the full EH123 construct, suggests that the EH domains might bind to Eps15’s own IDR, which has also been suggested previously^13,16,42^. Addition of Eps15_IDR_ to ^15^N EH123 indeed led to CSPs and increased ^15^N R_1ρ_ relaxation rates confirming this intra-molecular interaction (Fig. 5A). Our data recorded on ^15^N labeled Eps15_IDR_ show that EH123 binds to all phenylalanine containing regions of Eps15_IDR_, highlighting the promiscuity of EH123 also observed in the binding to Dab2_320–495_ (Fig. 5B). While one would expect that an NPF containing IDR, such as Dab2_320–495_, might replace Eps15_IDR_ from EH123 due to the preferred interaction of EH domains with NPF motifs compared to more promiscuous binding sites, this seems not to be the case. When both Eps15_IDR_ and Dab2_320–495_ are added to ^15^N labeled EH123, the spectrum and corresponding relaxation rates show signatures of binding to both proteins, although the individual contributions cannot be disentangled due to their similar interaction sites on EH123 (Fig. 5A). A similar behavior is observed when a sample containing all three proteins is detected from the side of ^15^N Eps15_IDR_ or ^15^N Dab2_320–495_. Only a few regions within the spectra of Eps15_IDR_ and Dab2_320–495_ slightly revert towards their unbound state when both EH123 and the respective other IDR (Dab2_320–495_ or Eps15_IDR_) are added (Fig. 5D, E), suggesting that both Eps15_IDR_ and Dab2_320–495_ can bind EH123 at the same time.

How is this possible when Dab2_320–495_ and Eps15_IDR_ occupy the same binding sites on EH123? Key to this question is likely the multivalency by which both IDRs interact with EH123, such that, while some motifs of Dab2_320–495_ might be displaced from EH123 in a dynamic fashion when Eps15_IDR_ is added—or vice versa—others maintain, thereby creating a dynamic trimeric (or even higher order) complex. Which of the motifs remain bound to EH123 and which get displaced is certainly a function of the individual motif’s affinities. This is easiest visualized as a dynamic equilibrium where motifs in Dab2 are competing with Eps15’s own IDR for binding to the EH domains, forming a complex and dynamic network (Fig. 7A, B). The fact that neither Dab2_320–495_ nor Eps15_IDR_ can effectively out-compete the other suggests that the strengths of the individual interactions between EH123 and Dab2_320–495_ or Eps15_IDR_ are not vastly different. The affinity between the EH domains and Eps15_IDR_ might be increased in native full-length Eps15, as a result of proximity effects. However, our phase separation experiments with full-length Eps15 and Dab2_320–495_ show recruitment of Dab2 into the Eps15 condensates, arguing for a physiological relevance of the interactions seen between Dab2_320–495_, the EH domains and Eps15_IDR_ and the competition effects observed.

While binding of EH domains to an intrinsically disordered linker of the same protein has been observed for the EH domain contained in EHD2^43^, the interaction network established by Eps15 is certainly remarkable, since so many interactions take place and compete with each other (Fig. 7). This has implications for all other binding partners of Eps15, whether binding to the EH domains or Eps15’s IDR, since any binding partner will be competing with these intra-molecular interactions. Indeed, the interactions observed between Eps15 EH domains and Eps15_IDR_ could constitute some kind of auto-inhibitory mechanism, such as recently described for WW domain proteins^44^, which could be (partially) released by other interaction partners, such as Dab2. In this context, it should be noted that the interactions between EH123 and Eps15_IDR_ could be both intra-molecular within one Eps15 molecule and inter-molecular between different Eps15 molecules. Indeed, Eps15-Eps15 interactions have previously been suggested to drive Eps15 phase separation in the context of clathrin-mediated endocytosis. For example, based on previous literature on the interaction between the EH domain of POB1 and DPF motifs^42^, which are also contained in Eps15_IDR_, interaction between Eps15 EH domains and Eps15_IDR_ has been suggested to be important for liquid-liquid phase separation^16^. While our molecular data show that not only DPF motifs, but also other F-rich protein regions are involved in this interaction, other interactions of similar strengths between Eps15 and its binding partners may drive this partner into the liquid-like droplets, such as it has been observed for FCHo1/2^16^ or ubiquitin^45^. Of note, phenylalanines have been identified as critical ‘stickers’ to promote weak interactions in liquid-liquid phase separation of IDPs^46^. Usually, they are thought to interact with phenylalanines in other IDPs, thereby creating a dynamic interaction network. In the case of Eps15, we have observed phenylalanines to contact small folded domains (EH domains)—interactions that may also drive Dab2_320–495_ into Eps15 droplets. In good agreement with this hypothesis, we observe that Dab2_320–495_, which does not form droplets on its own, also enters liquid-like droplets of Eps15, likely due to the weak multivalent network between the two (Fig. 7C). Therefore, while current literature points to Eps15 as the main initiator of condensate formation during the early phases of CME^16^ our data suggest that Dab2 may be recruited into such condensates also in the cellular context. Interestingly, even though Eps15’s IDR and Dab2_320–495_ seem to compete for the same interaction sites on EH123, the presence of Dab2_320–495_ had only negligible effects on the Eps15 condensates (Fig, 6 and Supplementary Figs. 22, 23) and even a large excess of Dab2_320–495_ did not dissolve Eps15 droplets (Supplementary Fig. 23). This behavior may make sense in the context of the early clathrin coated pit: Eps15 condensates are indeed thought to function as an initiator^16^, responsible to accumulate downstream client proteins that make endocytosis progress. Eps15 only moves away from the pit later in the process, when many other CLASPs/accessory proteins will have enriched at the pit^47,48^. It is thus possible that a much higher concentration of competitive interaction partners from different regions of Dab2, but also other NPF (and non-NPF) interaction partners are needed to affect phase separation or segregate these proteins out of Eps15 condensates. Indeed, it will be interesting to see whether the interactions between EH domains and Eps15_IDR_ are maintained also in the presence of proteins containing multiple NPF motifs, binding with higher affinities^18^, and what consequences this has on liquid-liquid phase separation at the endocytic pit and thus progression of productive endocytosis.

## Methods

### Cloning

Eps15-pmCherryN1 was a gift from Christien Merrifield (Addgene plasmid # 27696; http://n2t.net/addgene:27696; RRID: Addgene_27696)^48^. The three individual EH domains (EH1, EH2, EH3) and Eps15_IDR 761-896_ were cloned into the pET41c vector leading to constructs with a non-cleavable C-terminal His-tag. Full length Eps15, EH123, Eps15_IDR_, Eps15_IDR 481–581_, Eps15_IDR 569-671_ and Eps15_IDR 648–780_ were cloned into the pET28a vector with an N-terminal GB1 solubility tag pET28-6His-GB1. This leads to the expression of a TEV (tobacco etch virus) cleavable 6His-GB1-TEV site construct followed by the protein of interest. The gene of Dab2_320–495_ was purchased from Twist Bioscience and cloned into a pET28a vector with non-cleavable C-terminal His-tag. For the purpose of fluorescence labeling, a single cysteine mutant of Dab2_320–495_ was constructed using site-directed mutagenesis (Dab2_320–495 S328C_). Dab2_328–360_ and Dab2_358–390_ were cloned into a pET28a vector with an N-terminal GB1 solubility tag pET28-6His-GB1. The UniProt IDs of Eps15 and Dab2 used in this study are P42566 and P98082, respectively. The primer list for all cloning performed can be found below:

**Table:** 

**Restriction enzyme cloning**	
Eps15_120_Xho1_rv	GTG GTG CTC GAG CTC AGC TGC AGA GGT TCC
Eps15_215_Xho1_rv	GTG GTG CTC GAG TCT CTT AGA TGG TGG CAC CAA GGC
Eps15_310_Xho1_rv	GTG GTG CTC GAG TGG TGG AAT CAT TTC AGG AGT AAG AAC GTG
Eps15_671_Xho1_rv	AGT GCC TCG AGT TAA GTG CTT GAA GTG GCA AAA GGA TCA G
Eps15_780_Not1_rv	GTG GTG GCG GCC GCT CTT GTT GGA GTT CCG ATC TTT GGT GG
Eps15_896_Not1_rv	GTG GTG GCG GCC GCC CGT GCT TCT GAT ATC TCA GAT TTG CTG AG
Dab2_360_Not1_rv	GCA CTG CGG CCG CTT ATT GGG CCT CTT GCT TCC CG
Dab2_390_Not_rv	GCA CTG CGG CCG CTT AGG AAA ATC CGT TTT GCT CGC GC
Eps15_1_Nde1_fw	GAT ATA CAT ATG GCT GCG GCG GC
Eps15_121_Nde1_fw	GAT ATA CAT ATG CCA TGG GCT GTA AAA CCT GAA GAT AAG GC
Eps15_216_Nde1_fw	GAT ATA CAT ATG AAA ACG TGG GTT GTA TCC CCT GCA G
Eps15_481_Nde1_fw	GAT ATA CAT ATG CAC CTA CAA GAT TCA CAA CAG GAA ATT AGT TCA ATG C
Eps15_761_Nde1_fw	GATATA CATATG TCG GTC AAA AGT GAA GAT GAA CCC CC
Eps15_569_Nde1_fw	GTG CGC ATA TGT CTG GTG TGA CTG ATG AAA ATG AGG TG
Dab2_328_Nde_fw	GTG CGC ATA TGA GCA CCC CCT TGT CCA ATG G
Dab2_358_Nde_fw	GTG CGC ATA TGG AGG CCC AAG CGG GC
**Q5® Site-Directed Mutagenesis Kit (NEB)**	
Eps15-IDR1(481-581)_F	CTC GAG CAC CAC CAC CAC CAC C
Eps15-IDR1(481-581)_R	AAC AGC TGT AGT CAC CTC
Eps15-IDR1(648-780)_F	AAA GGT TCA GAT CCA TTT G
Eps15-IDR1(648-780)_R	CAT ATG ACC CTG GAA GTA C
**Site directed mutagenesis**	
Dab2_S328C_fw	CTA GCA GCT GTA CCC CCT TGT CCA ATG GTC C
Dab2_S328C_rv	CAA GGG GGT ACA GCT GCT AGA ACT ATT CTC CTT CTT CAT GGT ATA TC

### Protein expression and purification

The proteins were expressed in the *E. coli* Rosetta (DE3) strain and grown in LB medium with 30 mg/L Kanamycin and 30 mg/L Chloramphenicol at 37 °C. When the optical density (OD) at 600 nm was around 0.6–1, expression was induced with 1 mM isopropyl-β-D-thiogalactopyranoside (IPTG) and the expression was then continued at 20 °C overnight. For isotope labeling (^15^N, ^13^C) M9 minimal medium was used and supplemented with 1 g/L ^15^NH_4_Cl and/or with 2 g/L ^13^C-glucose.

Cells were lysed by sonication in lysis buffer (20 mM Tris, 150 mM NaCl pH 8, with Roche Ethylenediaminetetraacetic Acid (EDTA)-free protease inhibitor cocktail (Sigma-Aldrich Chemie GmbH)). Purification involved a two-step process: initial nickel purification followed by size-exclusion chromatography (SEC). The nickel column was equilibrated in lysis buffer before the filtered lysate was applied to a nickel column. The column was then washed with lysis buffer containing 20 mM imidazole, and the protein was eluted using lysis buffer supplemented with 400 mM imidazole. For all constructs expressed in the pET28-6His-GB1 vector, the eluted fraction, which contained the target protein (validated by SDS-PAGE and Coomassie staining), underwent overnight dialysis with 1 mg TEV protease at 4 °C in 500 mL of lysis buffer supplemented with 5 mM β-mercaptoethanol before proceeding to the SEC purification step. Proteins were further purified using a Superdex 75 or a Superdex 200 column, equilibrated in NMR buffer (50 mM Na-phosphate pH 6, 150 mM NaCl, and 2 mM dithiothreitol (DTT)). Fractions containing pure protein (validated by SDS-PAGE) were concentrated and frozen with final protein concentrations determined by absorbance at 280 nm using extinction coefficients determined by Expasy Protparam^49^.

### NMR spectroscopy

NMR experiments were measured at the Leibniz-Forschungsinstitut für Molekulare Pharmakologie (FMP), Berlin, Germany (^1^H frequencies of 600, 750, 900, 1200 MHz), and at the Institute of Structural Biology (IBS), Grenoble, France (^1^H frequencies of 600 MHz). The spectrometers were equipped with either room-temperature- (750 MHz) or cryo-probes (600, 900 MHz, 1200 MHz). All experiments were measured in NMR buffer (50 mM Na-phosphate pH 6, 150 mM NaCl, 2 mM DTT) at 25 °C. Addition of up to 5 mM CaCl_2_ did not affect our results. Spectra were processed with NMRPipe^50^, using qMDD^51^ for non-uniformly sampled assignment spectra, and analyzed with CCPN^52^. ^1^H-^15^N HSQC, TROSY-HSQC and triple resonance experiments were acquired using NMRlib^53^. The software TopSpin 3.5 and 4.4.1 (Bruker) were used for data acquisition.

### ^15^N, ^13^C Backbone assignment

Almost complete assignments could be obtained for all protein constructs. The assignment spectra of the ^15^N, ^13^C labeled EH1, EH2, EH3, Dab2_328–360_, and Dab2_358–390_ were acquired at a ^1^H frequency of 750 MHz. Assignment spectra of Eps15_IDR_, Eps15_IDR 480–581_, Eps15_IDR 569–671_, Eps15_IDR 648–780_, Eps15_IDR 761–896_, and Dab2_320–495_ were acquired at a ^1^H frequency of 600 MHz. Standard BEST-TROSY triple resonance experiments correlating CO, Cα, Cβ resonances (HNCO, HNCOCA, HNCA, iHNCA, HNCOCACB, iHNCACB)^54^ were acquired. The assignments were done in CCPN^52^ and then validated using MARS^55^. Secondary chemical shifts and secondary structure propensities^29^ were calculated using random coil values from refDB^56^. Overlapping the 3 TROSY-HSQC spectra of the individual EH domains (EH1, EH2, EH3) we transferred the ^1^H and ^15^N resonances to the TROSY-HSQC of the EH123 domain. The assignment of the different Eps15_IDR_ stretches was transferred to the spectrum of the full Eps15_IDR_ in a similar way. Dab2_320–495_ comprises many prolines, and some peaks within its ^1^H-^15^N HSQC spectrum could be attributed to appear due to cis/trans isomerization of neighboring prolines. We have not observed differences in binding due to cis/trans proline isomerization for the interactions tested and therefore all plots within the manuscript refer to the main conformational state of the protein.

### Titrations, ^15^N relaxation, and relaxation dispersion

Extraction of peak intensities (I) as well as ^1^H and ^15^N chemical shifts, were carried out from ^1^H-^15^N HSQC or BEST-TROSY-HSQC spectra. Combined chemical shift perturbations (CSPs) were calculated using

1 $${CSP}=\sqrt{{\left(\delta {}^{1}H\cdot 6.5\right)}^{2}+{\left(\delta {}^{15}N\right)}^{2}}$$

The specific concentrations used in the different titrations are indicated in the respective figures, with the percentage of partner indicating the molar ratio of partner protein:observed protein.

Residue specific *K*_d_ values were estimated by fitting the following equation to the CSPs as a function of concentration:

2 $$\Delta {CSP}=\Delta {{CSP}}_{\max }\cdot \frac{\left[{Dab}2\right]+\left[{EHx}\right]+{Kd}-\sqrt{{(\left[{Dab}2\right]+\left[{EHx}\right]+{Kd})}^{2}-4\cdot \left[{Dab}2\right]\cdot [{EHx}]}}{2\cdot [{Dab}2]}$$

Fitting was performed with Python.

^15^N R_1ρ_, R_1_ and {^1^H}-^15^N HetNOE relaxation rates^57^ were assessed at 600 MHz and 1200 MHz ^1^H Larmor frequency. The spin-lock field for the R_1ρ_ experiment was set to 1500 Hz or 2000 Hz for the 600 MHz and 1200 MHz magnets, respectively, and 6–7 delays, between 10 and 230 ms (for the disordered Dab2_320–495_ and Eps15_IDR_) and between 10 and 70 ms (for the folded EH domains) were used to sample the decay of magnetization. The relaxation rates were determined from an exponential fit to the peak intensity relative to the delay time. Errors of the fitted rates were derived from the experimental uncertainty. Inter scan delays were typically of 1.9 s. R_1_ was measured using 6–7 delays between 0 to 1.2 s with an inter-scan delay of typically 1.9 or 1.3 s. Inter scan delays of HetNOE experiments were 1 s.

Relaxation dispersion experiments^58^ were conducted at 600 and 900 MHz, employing 14 CPMG frequencies ranging from 31 to 1000 Hz, with a constant-time relaxation of 32 ms. R_2_ uncertainty in the CPMG experiments was estimated via Monte Carlo sampling from a normal distribution based on the experimental noise. The error bars represent the 15.9th and 84.1st percentiles of the resulting R_2_ distribution and were determined by the software ChemEx (https://github.com/gbouvignies/chemex). For the experiment with 100 µM Dab2_320–495_ in the presence of 10% EH2, 12 residues within the helix_N_ and the NPF motif (Val340, Asp341, Gln345, Gln346, Gln349, Ser351, Thr354, Lys356, Phe366, Phe 398, Val399, Asp426) were fit globally using a 2 site exchange model with the software ChemEx (https://github.com/gbouvignies/chemex). An exchange rate of 149 ± 13 s^−1^ and a percentage bound of 3 ± 0.3% were obtained from the fit.

### Conformational ensemble of Dab2_320–495_

A conformational ensemble was calculated from Dab2_320–495_ and Dab2_328-360_ using a combination of the statistical coil generator, flexible meccano^24^, and the genetic algorithm ASTEROIDS^25^. From a statistical coil ensemble of 10,000 conformers, 200 conformations that together best described the H_N_, N, CO, Cα and Cβ chemical shifts of the proteins were selected. A new ensemble of 8500 conformers was generated using the Φ and ψ angles from the previous selection and supplemented with 1500 conformations from the previous pool of conformers. A new selection of 200 conformations was performed on the new pool. This iteration was repeated 4 times. Ensemble-averaged chemical shifts were generated using SPARTA^59^, and secondary chemical shifts were calculated based on RefDB^56^.

### Structures of the individual EH domains

The structures that were used to plot the CSPs for EH2 and EH3 are the PDB codes 1FF1^31^ and 1C07^32^, respectively. For EH1, we use an AlphaFold2^34,60^ prediction since at the time of writing no *human* Eps15 EH1 structure was deposited in the PDB. The CSPs of each EH domain with 100 μM Dab2_320–495_ (100%) were plotted onto the structures. The linear gradient is from 0%, which represents no CSP, up to 100%, which is the highest CSP observed at 0.75 ppm. The structures were visualized using Pymol.

### Protein labeling with fluorescent dyes

Protein labeling was performed using malemeide dyes; AZDye488 (Vectorlabs, CA, USA), for Dab2_320–495 S328C_ and AZDye594 (Vectorlabs, CA, USA) for Eps15 at random cysteine side chains, essentially as described previously^61,62^. After adding 10 mM DTT overnight to fully reduce the proteins, they were dialyzed into a phosphate-buffer (50 mM Na-phosphate pH 7, 150 mM NaCl) for 2 × 1 h at room temperature. At least 5 times molar excess of the dyes was used for the labeling reaction, which, after mixing, contained a maximum of 10% V/V DMSO. The protein/dye mixtures were allowed to react for 1 h at room temperature and incubated overnight at 4 °C. To stop the reaction, 10 mM of DTT were added before injecting the mixtures on a SEC70 or 650 equilibrated in NMR buffer to remove any unconjugated dye.

### Protein droplets and microscopy imaging

All droplet formation assays were performed in a buffer of 50 mM Na-phosphate pH 6, 150 mM NaCl, and 2 mM DTT, 3% w/v PEG8000, using different ratios of Eps15 (0.5 µM to 10 µM) and Dab2_320–495_ (0 µM to 140 µM). For both proteins, a ratio of 10:1 of non-labeled: fluorescently labeled protein was used. A total sample volume of 20 µL was placed in 8-well polystyrene chambers with 1.5 borosilicate coverglass (Nunc Lab-Tek). Epi-fluorescence imaging was performed with a Nikon Ti Eclipse microscope with a 60x (PLAN APO, NA:1.40, WD 0.13 mm) oil immersion objective. The setup was controlled by the imaging software NIS (Nikon). Confocal microscopy was performed with the LSM780, and the setup was controlled by Zeiss Zen Black software. Dab2_320–495 S328C_ AZDye488 (Ex.: 488 nm; EmF.: 490–535 nm) and Eps15 AZDye594 (Ex.: 561**;** EmF.: 569–631 nm) were imaged. A PL APO DIC M27 63 × /1.40 NA oil objective (Carl Zeiss Microscopy) at a zoom factor of 1 or 3 and a line average of 1 was used, acquiring images of 512 × 512/1024 × 1024 pixels, respectively. Microscopy images were imported and analyzed using ImageJ/Fiji^63^. The intensity ratio of the droplets versus background was calculated by manually selecting droplets and comparing the intensity to an identical area of the background. The final ratio was calculated as an average over all droplets picked across the different images for each condition. The number and size of the droplets were determined by applying an Otsu threshold to each image. The number of droplets was counted in each individual image for each condition, while the size of the droplets was calculated as an average of droplet size across all images of each condition. For the determination of the droplet size and count as well as the partitioning of Eps15 and Dab2_320–495_ into droplets, images at a zoom factor of 1 (512 × 512 pixels) were used, and the analyses from ImageJ/Fiji were plotted and visualized using Python. The number of images included in the analysis for each condition can be found in Fig. 6. The images were acquired at different spatial positions of the same samples, respectively.

### Reporting summary

Further information on research design is available in the Nature Portfolio Reporting Summary linked to this article.

## Supplementary information

Supplementary Information

Reporting Summary

Transparent Peer Review file

## Source data

Source data

## Data Availability

All study data are included in the article, supporting information, the Source Data files and/or can be obtained from the corresponding author upon request. The chemical shift assignments generated in this study have been deposited in the Biological Magnetic Resonance Data Bank (BMRB) under the accession numbers 52613 (Dab2_320–495_), 52866 (Eps15_IDR 481-581_), 52864 (Eps15_IDR 569-671_), 52863 (Eps15_IDR 648-780_), and 52867 (Eps15_IDR 761-896_), respectively. The NMR and imaging statistics generated in this study are provided in the Source Data files. The PDB entries of EH2 and EH3 used in this work are: 1FF1 (EH2) and 1C07 (EH3).  Source data are provided with this paper.
