# Supplementary material for: Promiscuous and multivalent interactions between Eps15 and partner protein Dab2 generate a complex interaction network
Source: Nat Commun. 2025 Aug 21;16:7783. doi: 10.1038/s41467-025-63090-1 (PMC12370899; doi:10.1038/s41467-025-63090-1)
Supplement: Supplementary file 1 — Supplementary Information [file 41467_2025_63090_MOESM1_ESM.pdf]

Supplementary Information

Promiscuous and multivalent interactions between Eps15 and partner protein Dab2 generate a complex interaction network.

Authors

Papagiannoula[#], Vedel[1#], *et al.*

[#]equal contribution

*milles@fmp-berlin.de

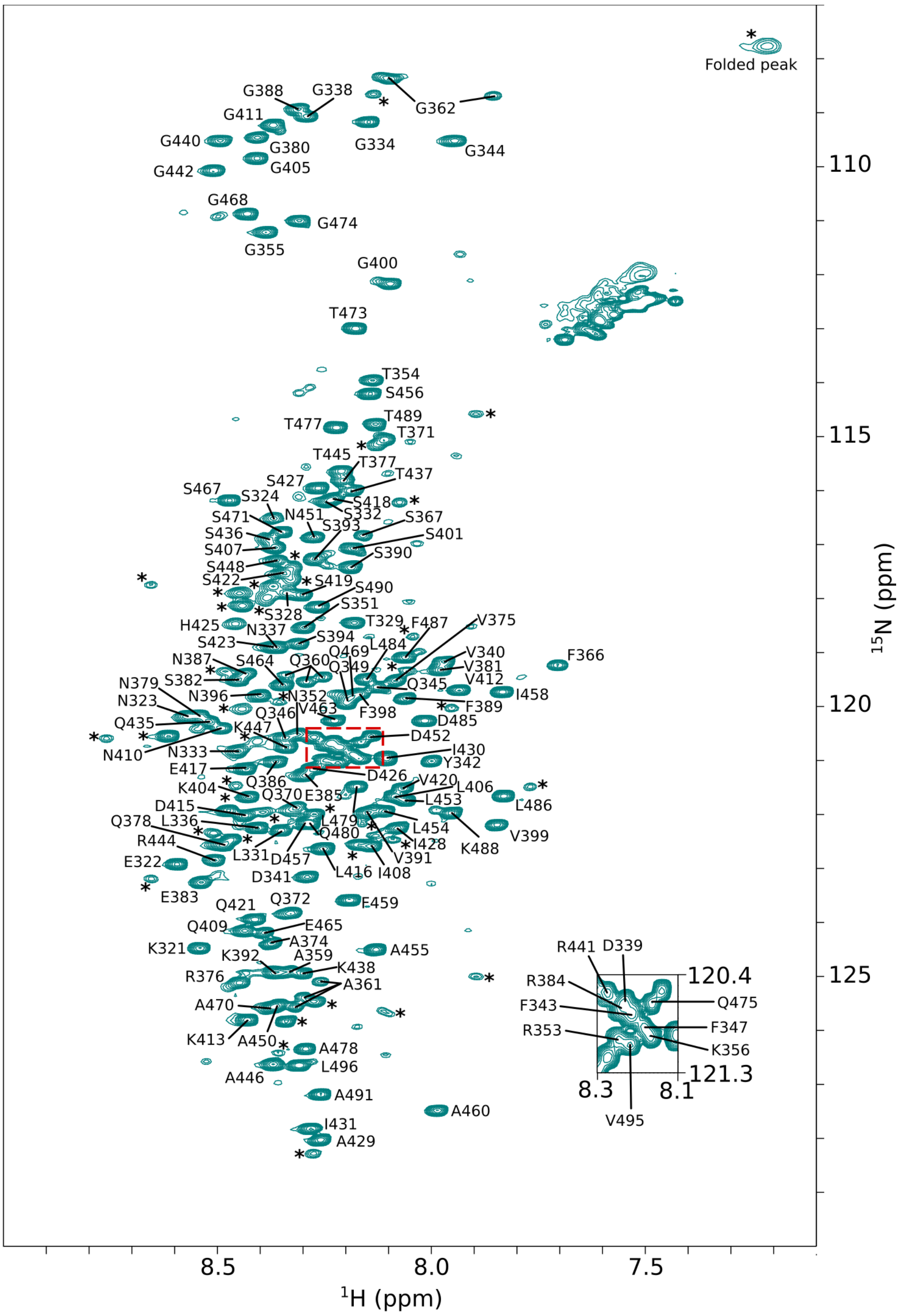

Supplementary Figure 1. Assignment of Dab2$_{320\text{-}495}$. The ^{1}H-^{15}N HSQC spectrum of Dab2$_{320\text{-}495}$ showing the backbone resonance assignments as one letter amino acid code. The region marked with red dashed lines is shown as a zoom inside the spectrum. Some residues, e.g. G362, give rise to multiple resonances likely due to proline cis/trans isomerization. Only the main state is uploaded to BMRB (52613). Unassigned peaks are marked with asterisks. 86% of the non-proline residues have been assigned.

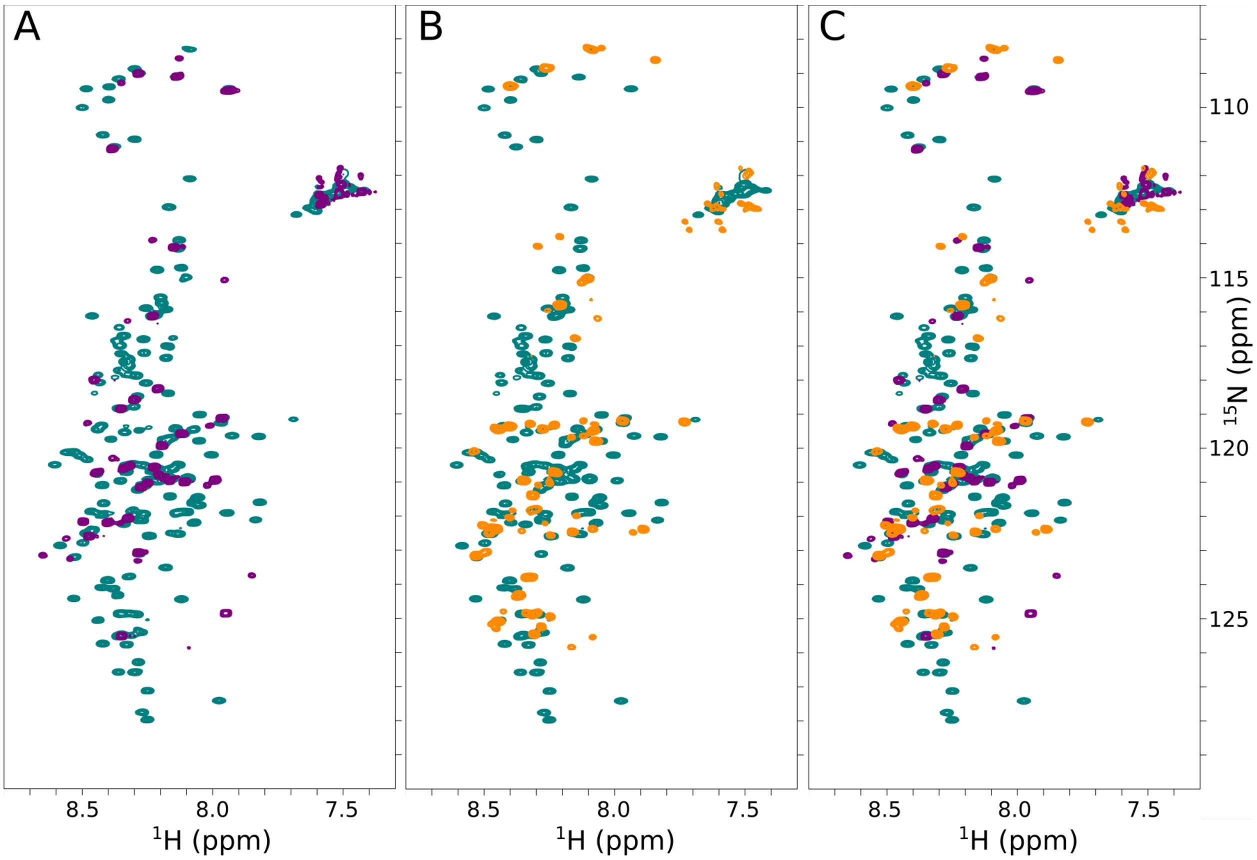

Supplementary Figure 2. Overlay of ^{1}H-^{15}N HSQC spectra of Dab2$_{320\text{-}495}$ (teal), Dab2$_{328\text{-}360}$ (purple) and Dab2$_{358\text{-}390}$ (orange). (A) Dab2$_{328\text{-}360}$ spectrum overlaid onto the spectrum of Dab2$_{320\text{-}495}$. **(B)** Dab2$_{358\text{-}390}$ spectrum overlaid onto the spectrum of Dab2$_{320\text{-}495}$. **(C)** Spectra of Dab2$_{328\text{-}360}$ and Dab2$_{358\text{-}390}$ overlaid onto the spectrum of Dab2$_{320\text{-}495}$.

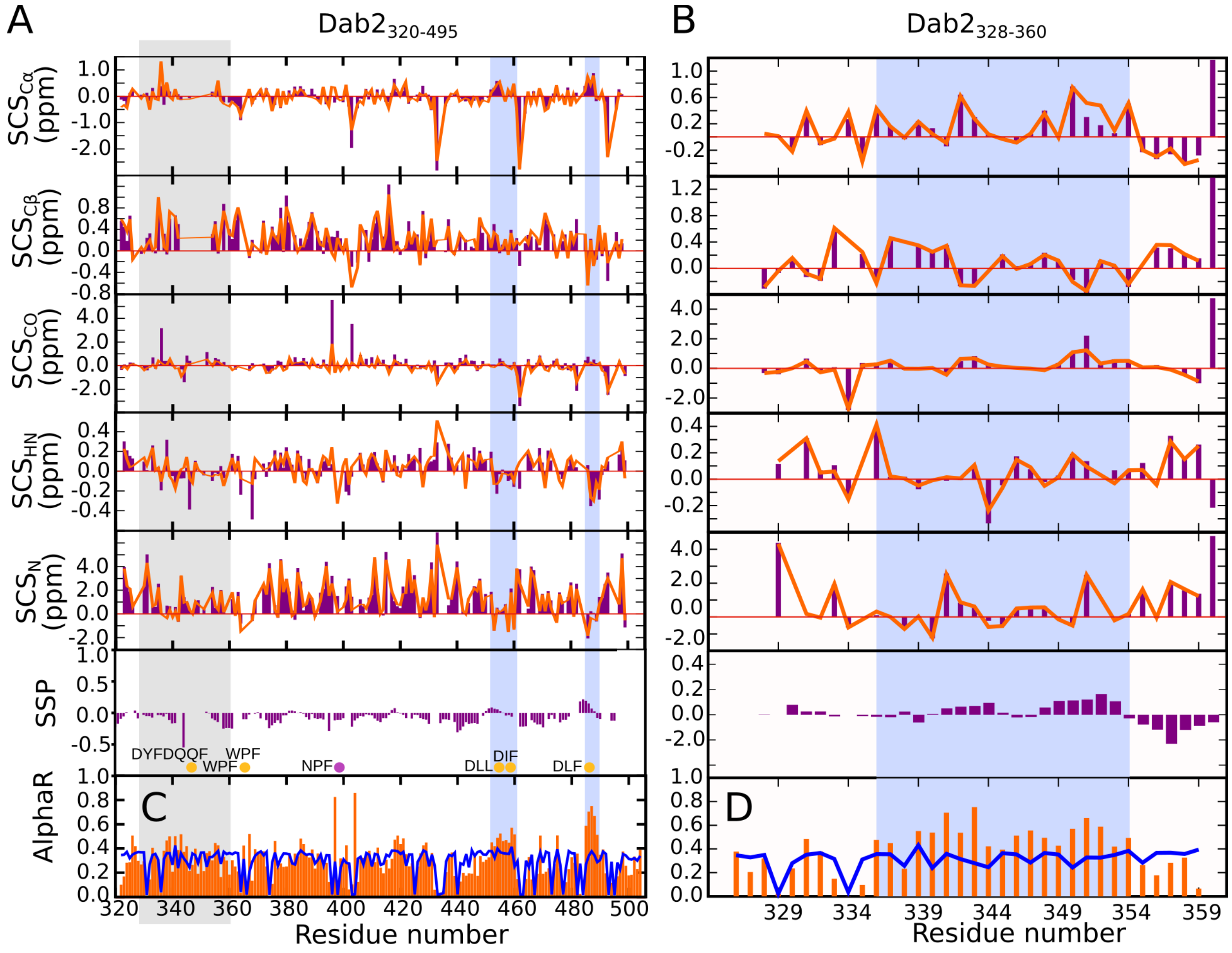

Supplementary Figure 3. Secondary chemical shifts and secondary structure propensities of (A) Dab2$_{320\text{-}495}$ and (B) Dab2$_{328\text{-}360}$. SCSs (purple bars) were calculated with respect to random coil chemical shifts from RefDB[1]. Secondary chemical shifts (SSPs)[2] (purple bars) were calculated based on Cα and Cβ chemical shifts. An SSP value of 1 reflects a fully formed helix, a value of -1 reflects a fully extended (β-strand) conformation. Back calculation of SCSs and SSPs on the basis of the ASTEROIDS ensemble is shown as orange lines above the experimental values in purple. An ASTEROIDS ensemble of Dab2$_{320\text{-}495}$ **(C)** and Dab2$_{328\text{-}360}$ **(D)** illustrate a mild increase in helical conformation (AlphaR, $\phi < 0°$, $-120° < \psi < 50°$)[3] (orange bars) as compared to random coil (blue line), from residues 338 to 354 approximately and additional helical sampling around residues 450 to 455 as well as around 480. Regions with increased helical propensity are highlighted in blue. The protein region in Dab2$_{320\text{-}495}$ for which the Dab2$_{328\text{-}360}$ construct was generated is highlighted in gray in panels A and C.

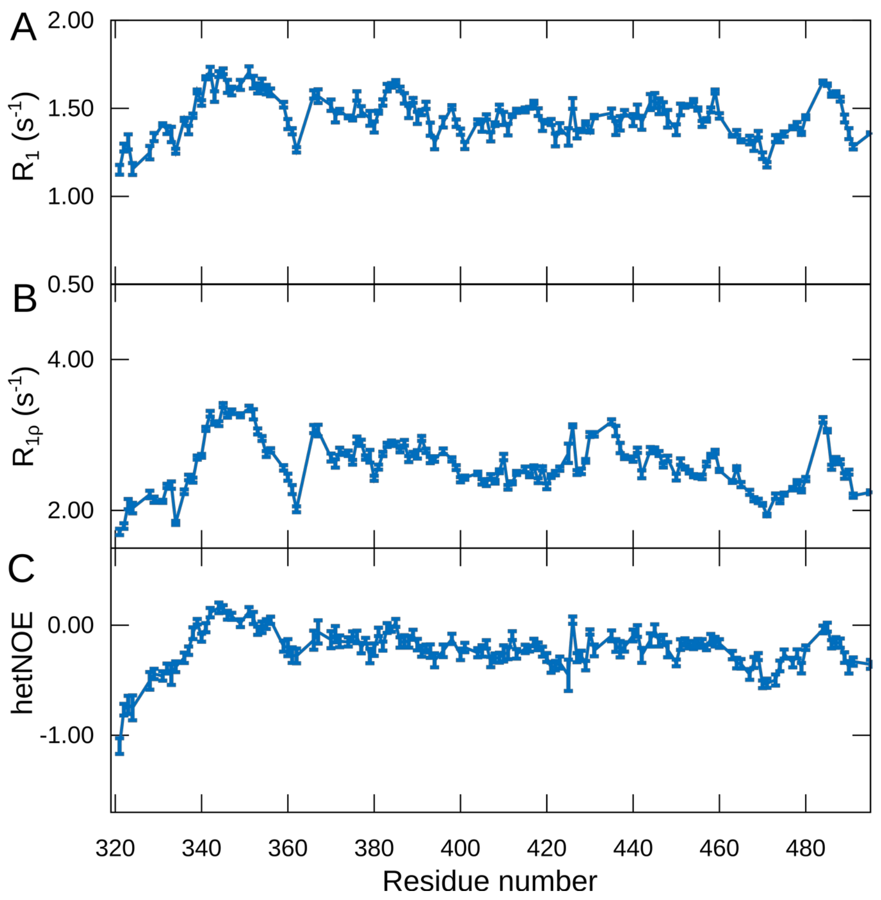

Supplementary Figure 4. ^{15}N backbone relaxation of Dab2$_{320\text{-}495}$. (A) ^{15}N R_1 relaxation, **(B)** ^{15}N $R_{1\rho}$ spin relaxation, **(C)** {^{1}H}-^{15}N HetNOE of Dab2$_{320\text{-}495}$. The experiments were recorded on a 270 µM (R_1) and 100 µM ($R_{1\rho}$ and hetNOE) Dab2$_{320\text{-}495}$ sample at a ^{1}H frequency of 600 MHz. Errors were derived from the experimental uncertainty.

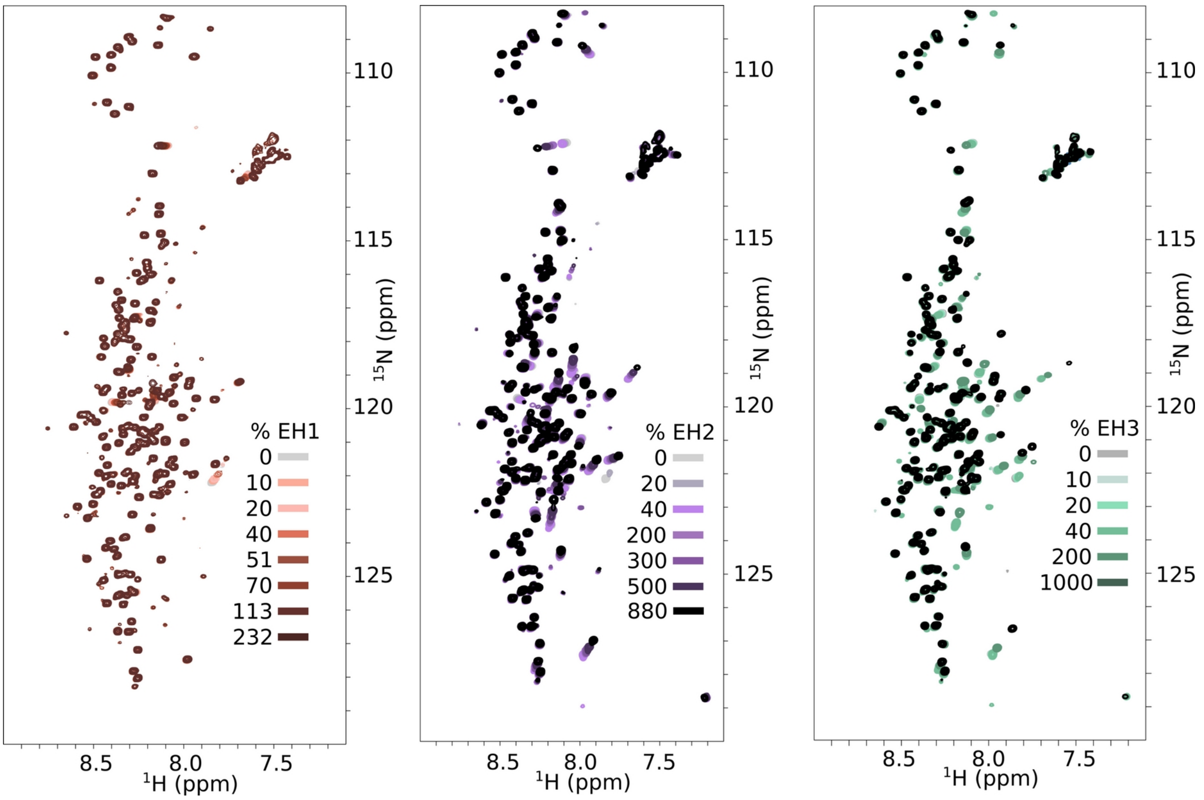

Supplementary Figure 5. Interaction of ^{15}N Dab2$_{320\text{-}495}$ with EH1, EH2, and EH3. ^{1}H-^{15}N HSQC spectra of Dab2$_{320\text{-}495}$ alone and in the presence of increasing concentrations of EH1 (left), EH2 (middle) and EH3 (right). Color codes of the different spectra are indicated in the figure. The concentration of Dab2$_{320\text{-}495}$ was kept constant at 100 µM throughout the titration.

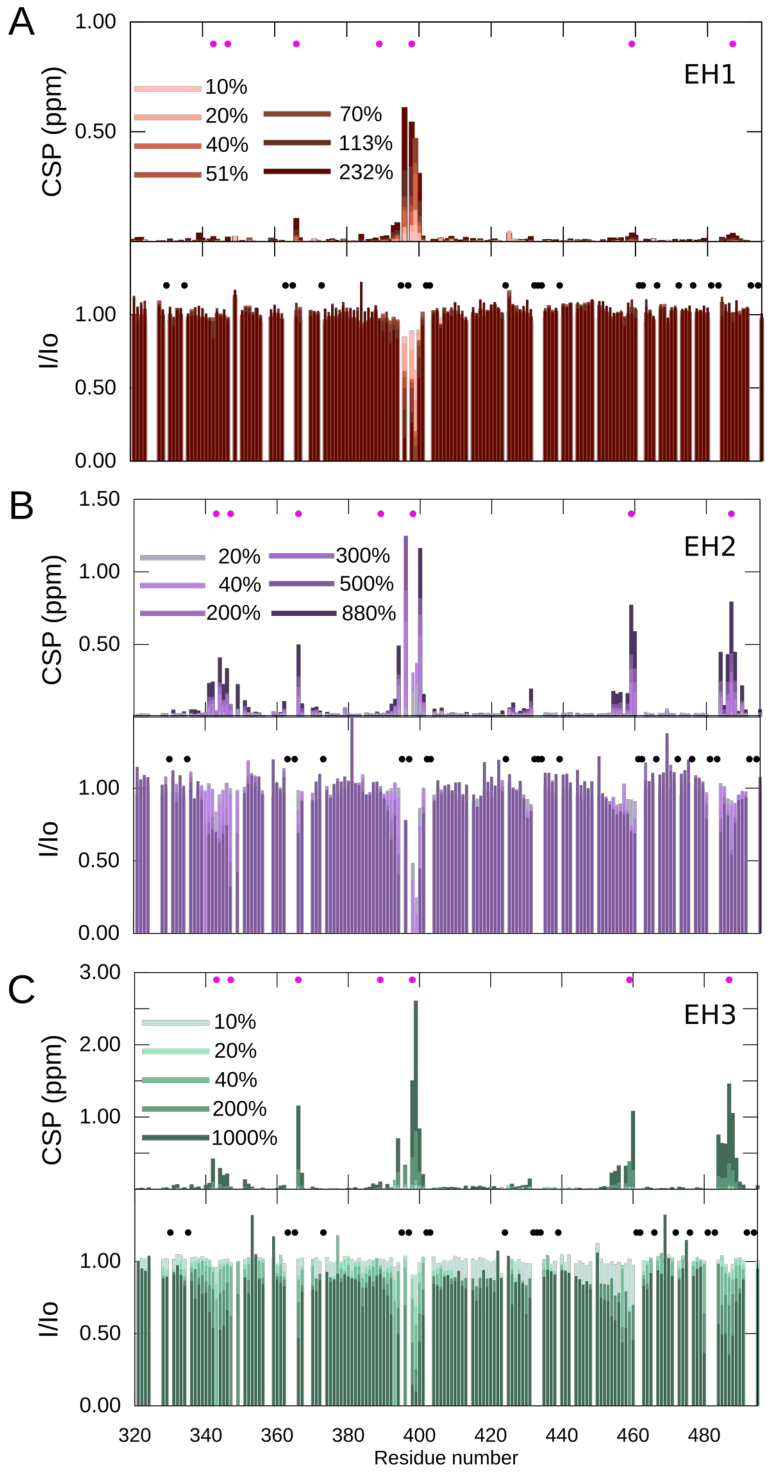

Supplementary Figure 6. Interaction of Dab2$_{320\text{-}495}$ with the individual EH domains. Top: CSPs extracted from Dab2$_{320\text{-}495}$ ^{1}H-^{15}N HSQC spectra in the presence of increasing concentrations of EH1 **(A),** EH2 **(B)**, EH3 **(C)** (see also Figure 1D, E, F, respectively). Bottom: Intensity ratio calculated from peak intensities extracted from Dab2$_{320\text{-}495}$ ^{1}H-^{15}N HSQC spectra in the presence of various concentrations of EH1 (A), EH2 (B), EH3 (C) versus the absence of interaction partner. Color legends are displayed in the respective plots. Filled pink circles denote positions of phenylalanines and filled black circles denote positions of prolines.

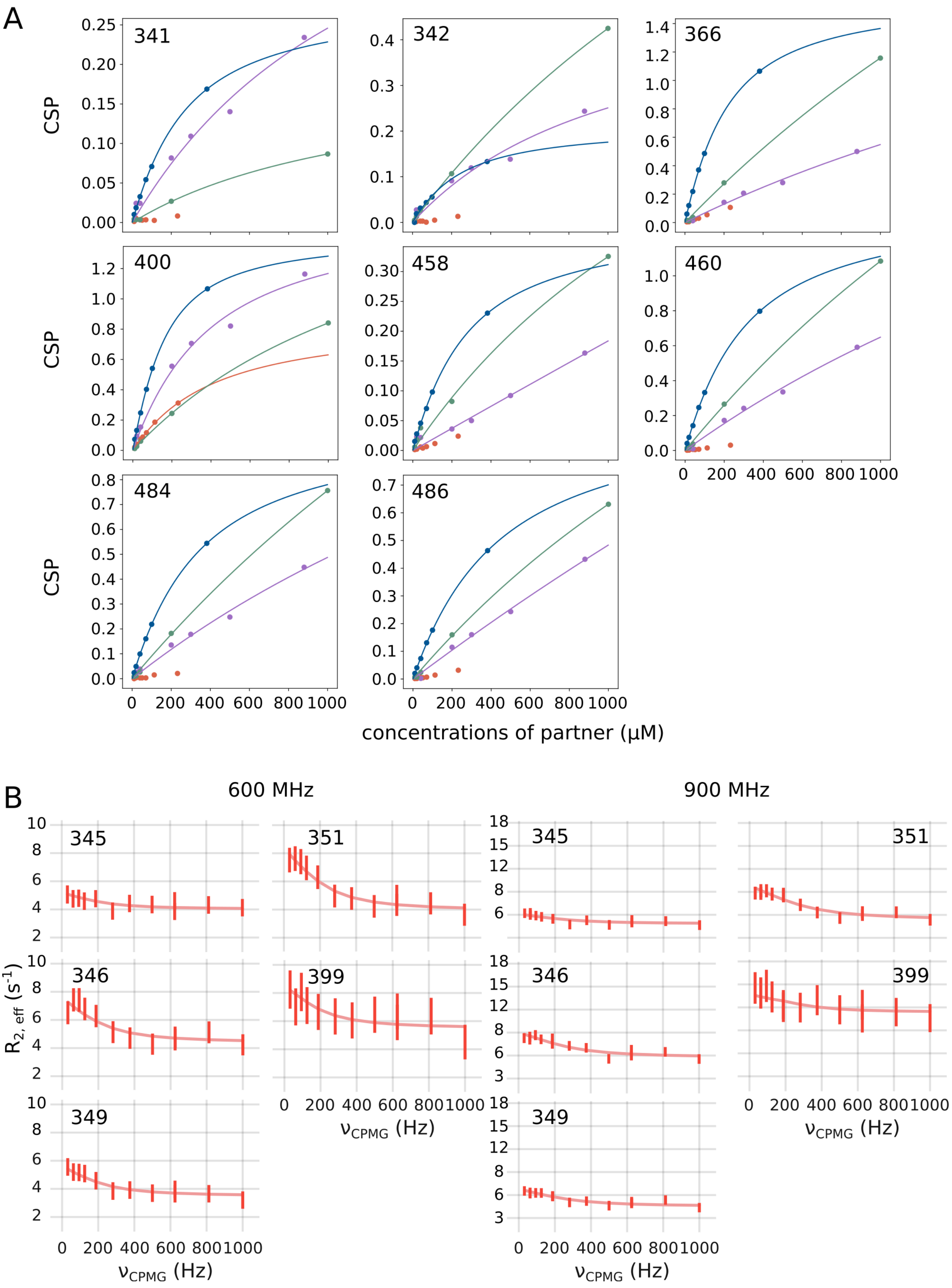
A
CSP
341
342
366
400
458
460
484
486
concentrations of partner (μM)
B
600 MHz
900 MHz
345
351
346
399
349
$R_{2, eff}$ (s^{-1})
ν_{CPMG} (Hz)

Supplementary Figure 7. Affinities between the Dab2$_{320\text{-}495}$ NPF motif and EH domains. (A) CSPs were calculated from ^{1}H and ^{15}N chemical shifts at increasing concentrations of EH1, EH2, EH3, or EH123 and plotted against these concentrations (filled points, blue: EH123, brown: EH1, purple: EH2, green: EH3). The data were then fit with a simple binding model (see methods, solid line). Affinities extracted from the fit can be found in Supplementary Table 1. **(B)** CPMG relaxation dispersion curves of five Dab2$_{320\text{-}495}$ residues in the presence of EH2. Dispersion is visible at the NPF motif (residue 399) and around the transient helix at the N-terminus of Dab2$_{320\text{-}495}$ (345-351). The data were fitted with a global fit across 12 residues in the helical region and the NPF motif (residues Val340, Asp341, Gln345, Gln346, Gln349, Ser351, Thr354, Lys356, Phe366, Phe398, Val399, Asp426) and resulted in an exchange rate k_{ex} of 149 ± 13 s^{-1} and a percentage of bound Dab2$_{320\text{-}495}$ of 3.3 ± 0.3%. The data were acquired at a concentration of 100 μM Dab2$_{320\text{-}495}$ with 10% of EH2, resulting in a K_D of 196 μM estimated from the CPMG data and in rough agreement with the chemical shift perturbations that suffer from intermediate exchange line broadening around the NPF motif. The data were recorded at ^{1}H frequencies of 600 and 900 MHz. R_2 uncertainty was estimated via Monte Carlo sampling from a normal distribution based on the experimental noise. The error bars represent the 15.9th and 84.1st percentiles of the resulting R_2 distribution. The fitting results from the CPMG data are summarized in Supplementary Table 2.

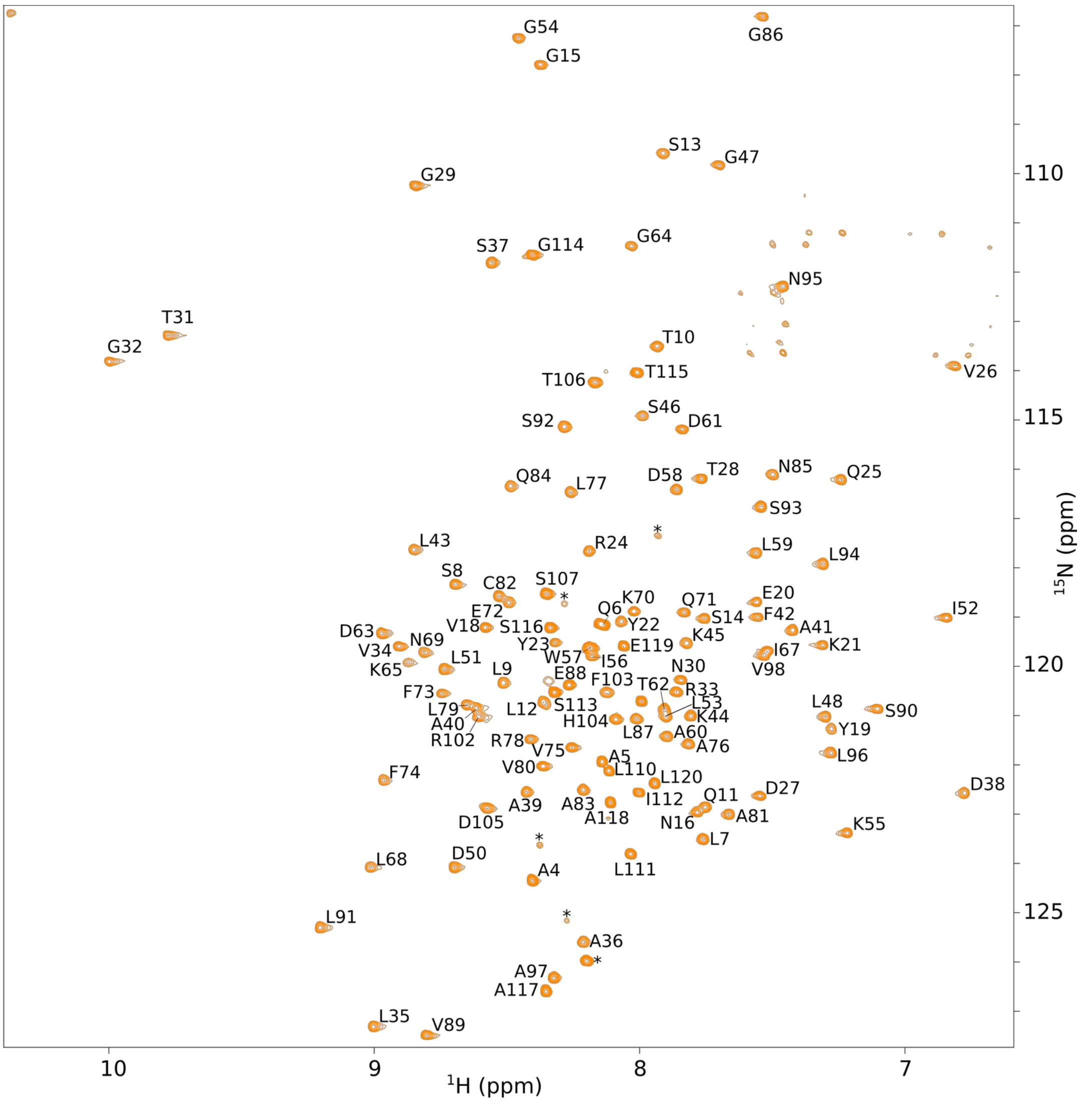

Supplementary Figure 8. Assignment of EH1. ^{1}H-^{15}N TROSY-HSQC spectrum of EH1, showing the backbone resonance assignments as one letter amino acid code. Unassigned peaks are marked with an asterisk. The peak at a ^{15}N chemical shift around 106 ppm and a ^{1}H chemical shift of around 10.2 ppm corresponds to a tryptophan side chain that has been aliased in the spectrum. 76% of all residues have been assigned.

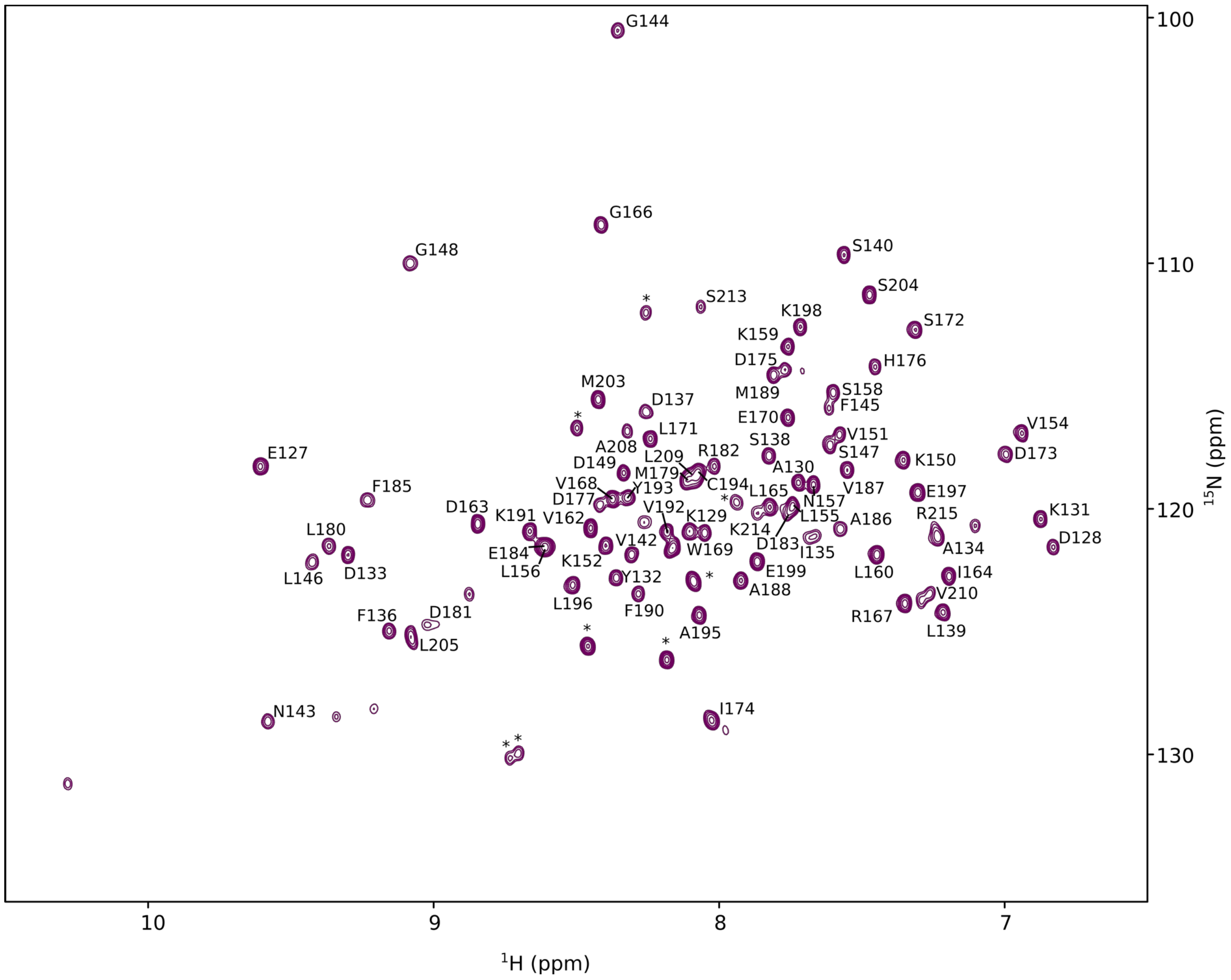

Supplementary Figure 9. Assignment of EH2. ^{1}H-^{15}N TROSY-HSQC spectrum of EH2, showing the backbone resonance assignments as one letter amino acid code. Unassigned peaks are marked with an asterisk. The peak at a ^{15}N chemical shift around 130 ppm and a ^{1}H chemical shift of around 10.2 ppm corresponds to a tryptophan side chain. 75% of all residues have been assigned.

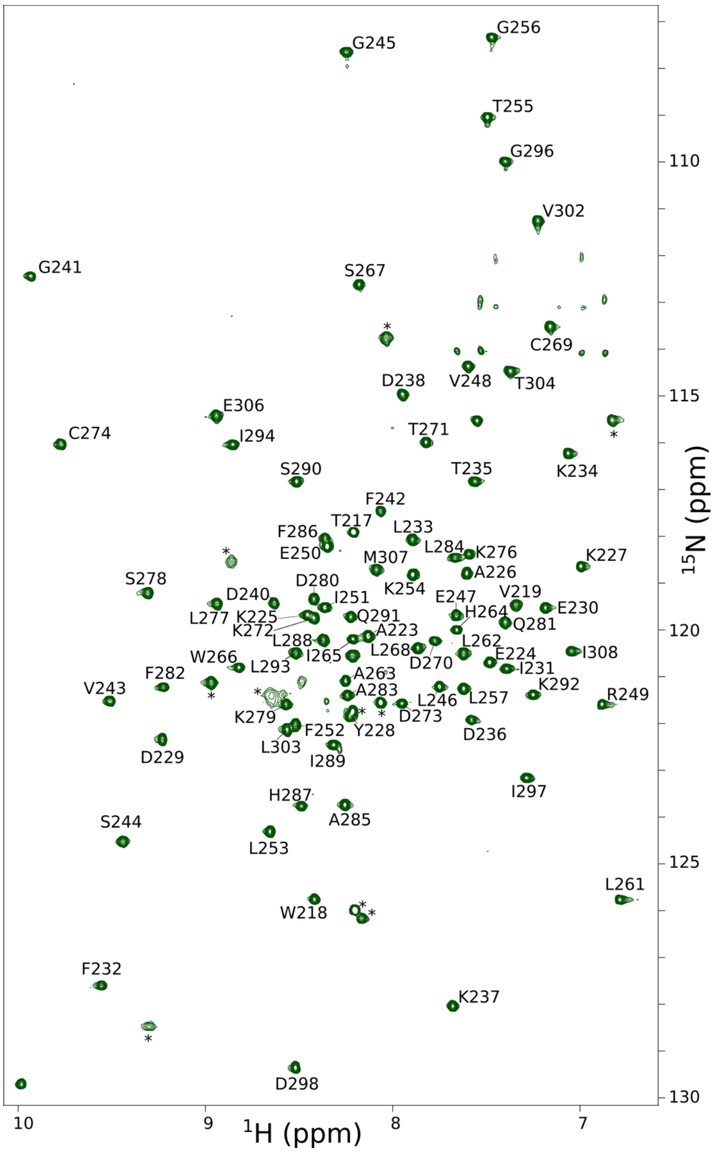

Supplementary Figure 10. Assignment of EH3. ^{1}H-^{15}N TROSY-HSQC spectrum of EH3, showing the backbone resonance assignments as one letter amino acid code. Unassigned peaks are marked with an asterisk. The peak at a ^{15}N chemical shift around 130 ppm and a ^{1}H chemical shift of around 10 ppm corresponds to a tryptophan side chain. 75% of all residues have been assigned.

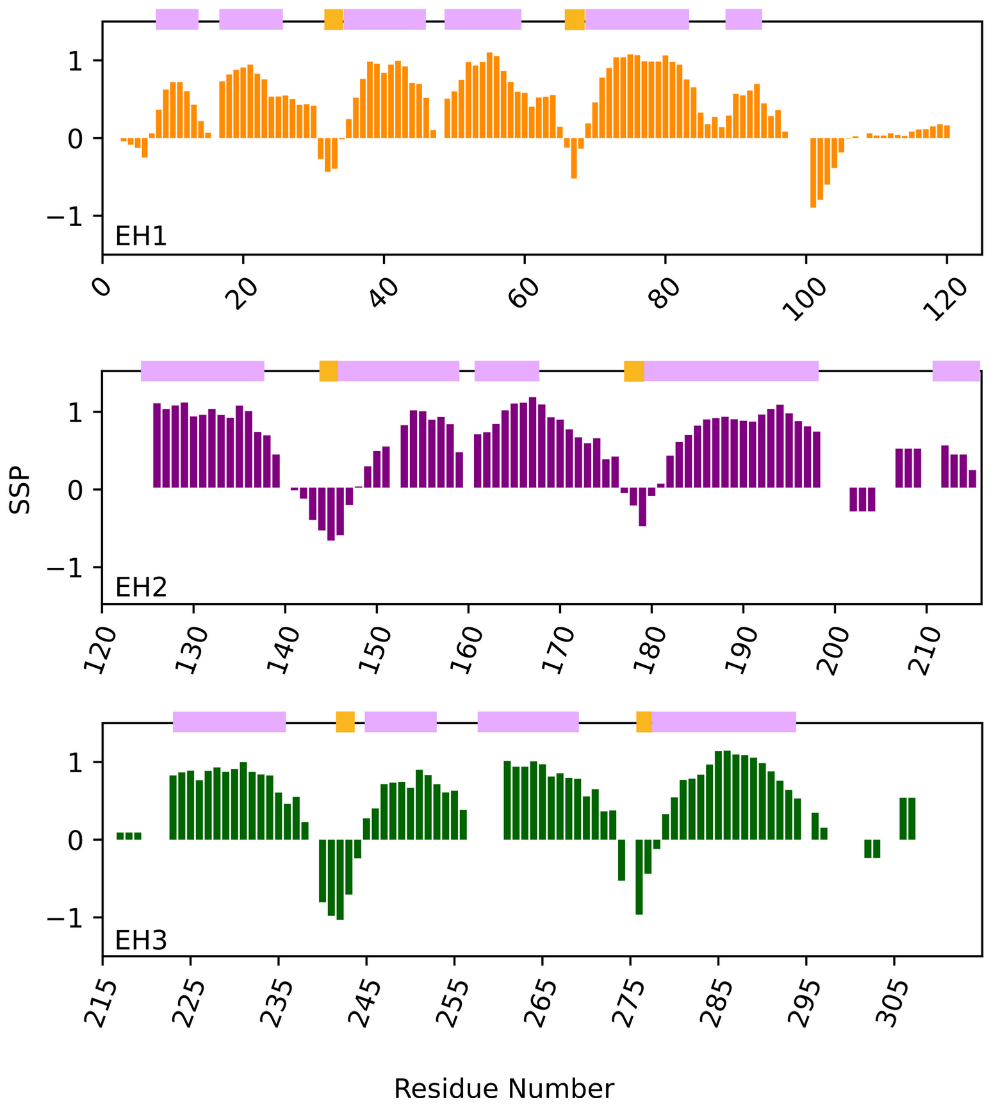

Supplementary Figure 11. Secondary structure analyses of the individual EH domains. SSPs of EH1 (top), EH2 (middle) and EH3 (bottom). SSPs[2] were calculated based on Cα and Cβ chemical shifts. A value of 1 reflects a fully formed helix, a value of -1 reflects a fully extended (β-strand) conformation. The pink boxes represent α-helical conformations and the yellow boxes represent β-sheets as observed in the respective PDB structures 1QJT[4] (*mouse* EH1), 1FF1[5] (*human* EH2), 1C07[6] (*human* EH3), showing that the assignments are in agreement with the already published structures.

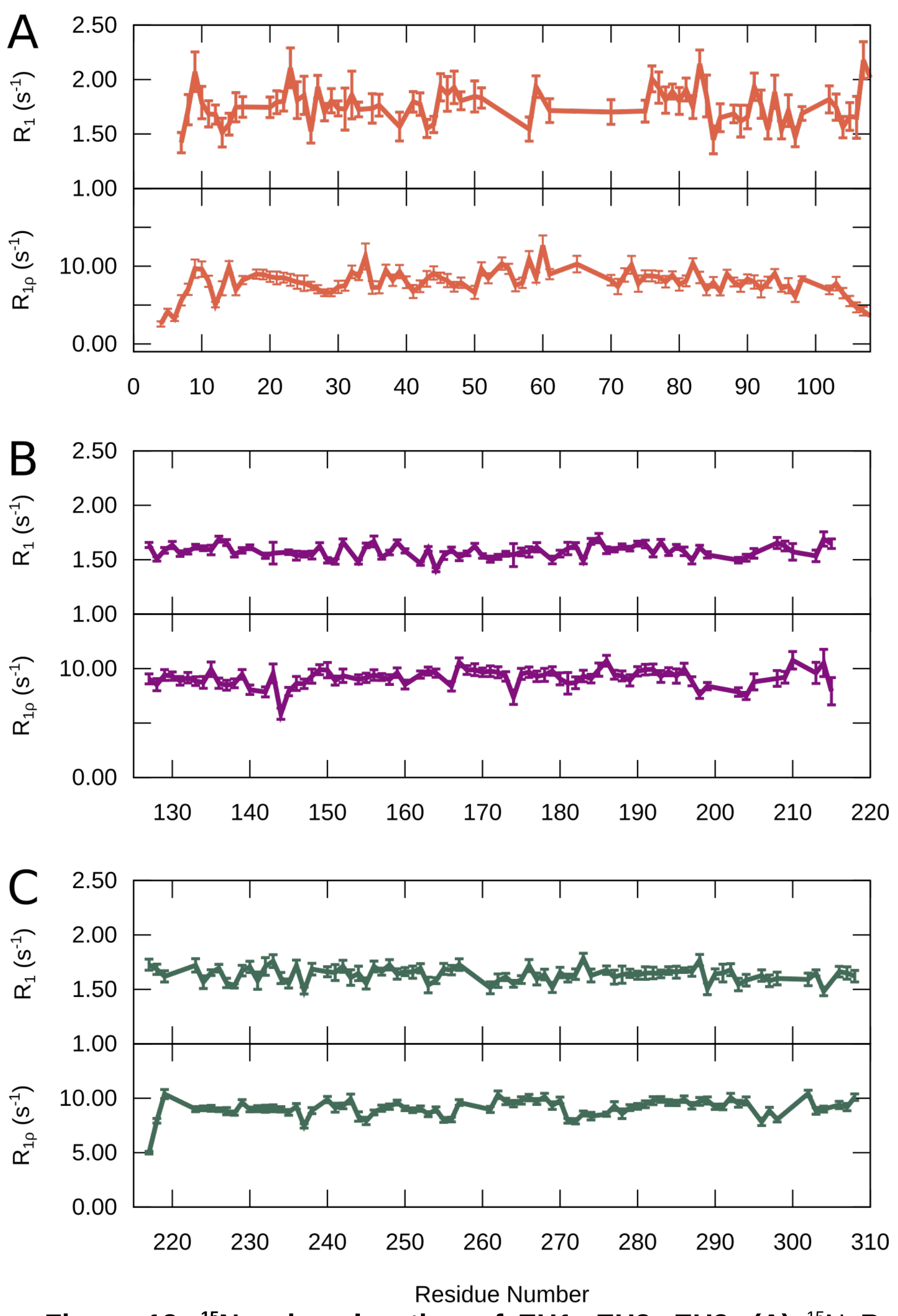

Supplementary Figure 12. ^{15}N spin relaxation of EH1, EH2, EH3. (A) ^{15}N R_1 and $R_{1\rho}$ spin relaxation of 100 μM EH1. **(B)** ^{15}N R_1 and $R_{1\rho}$ spin relaxation of 100 μM EH2. **(C)** ^{15}N R_1 relaxation of 576 μM EH3 and ^{15}N $R_{1\rho}$ spin relaxation of 100 μM EH3. The experiments were all recorded at a ^{1}H frequency of 600 MHz. Errors were derived from the experimental uncertainty.

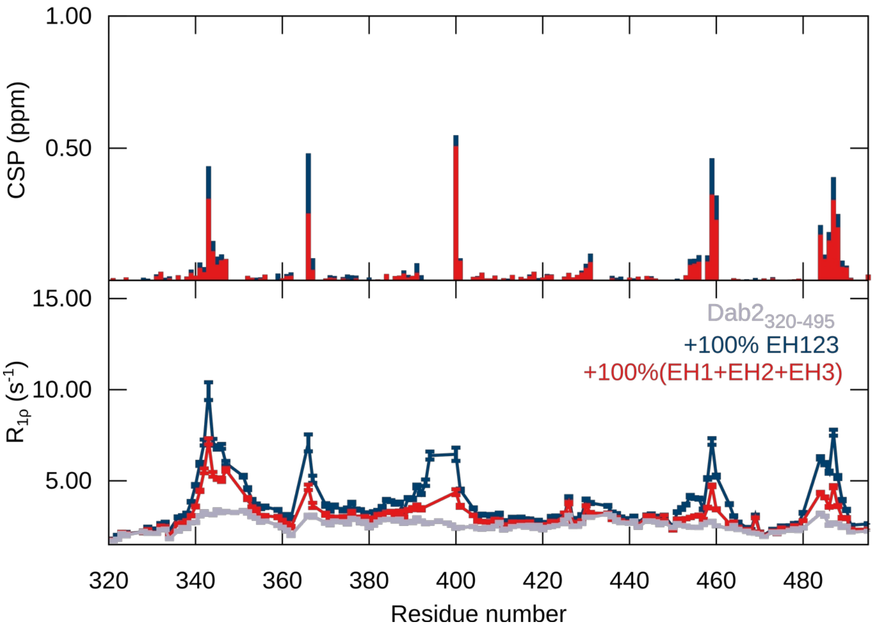

Supplementary Figure 13. Interaction of Dab2$_{320\text{-}495}$ with EH123 and EH1+EH2+EH3. CSPs of 100 µM Dab2$_{320\text{-}495}$ in the presence of 100% EH123 (Dark blue) or 100% of each of the individual EH1, EH2, EH3 domains (red). ^{15}N $R_{1\rho}$ relaxation rates of 100 µM Dab2$_{320\text{-}495}$ alone (gray) and in the presence of 100 µM EH123 (dark blue) or EH1 + EH2 + EH3, each at 100 µM (red). Errors of relaxation rates were derived from the experimental uncertainty.

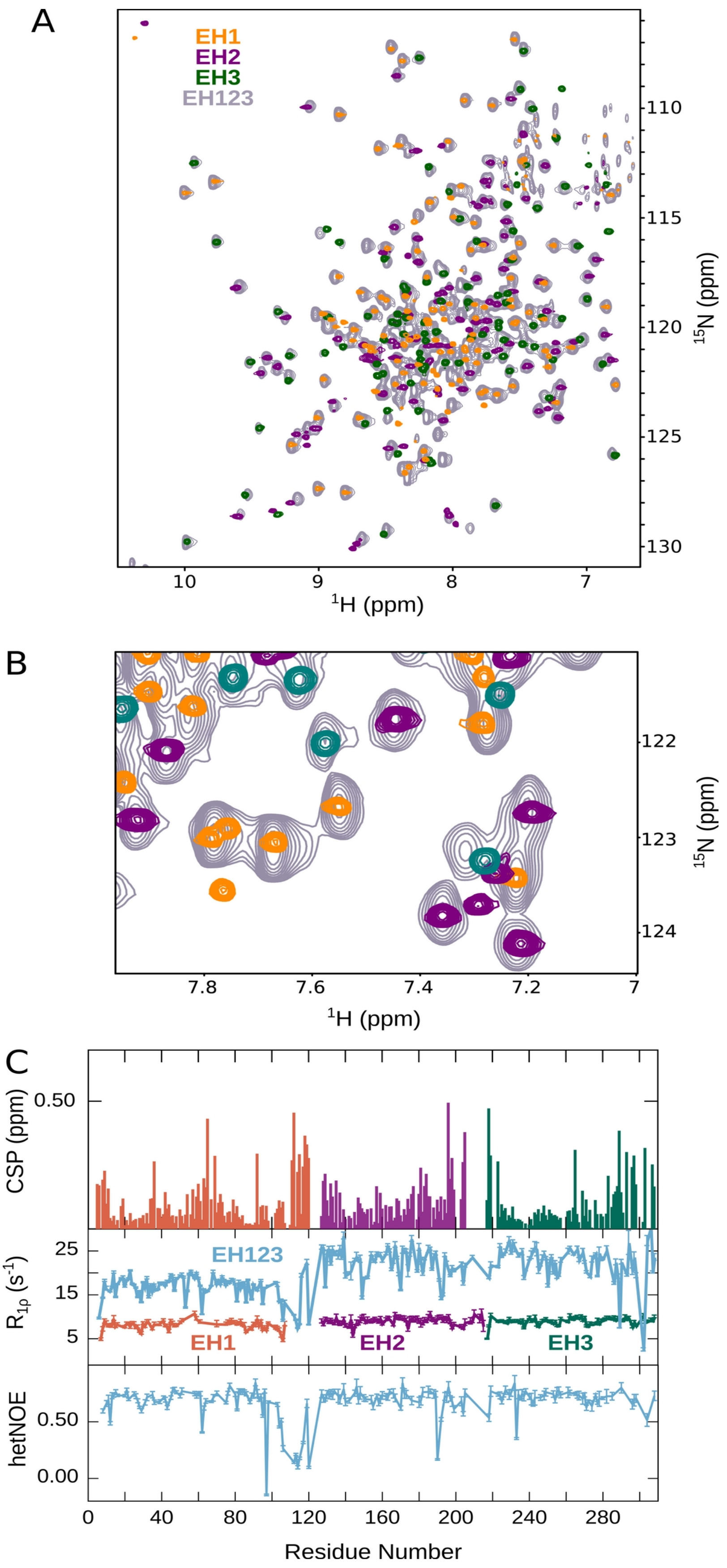

Supplementary Figure 14. EH2 and EH3 tumble together within EH123. (A) Overlay of ^{1}H-^{15}N TROSY-HSQC spectrum of EH123 (gray) and EH1 (orange), EH2 (purple), EH3 (green). **(B)** Zoom

into the ^{1}H-^{15}N TROSY-HSQC spectrum of A. **(C)** CSPs (top) calculated between the ^{1}H-^{15}N TROSY-HSQC of the ^{15}N EH123 domain and those of EH1 (orange), EH2 (purple), EH3 (green). ^{15}N $R_{1\rho}$ spin relaxation (middle) of EH123 (light blue) and EH1 (orange), EH2 (purple), EH3 (green). $\{^{1}H\}$-^{15}N HetNOE (bottom) of EH123 (light blue). The experiments were recorded at a ^{1}H frequency of 600 MHz. The concentration of EH123 is 260 µM. Errors of relaxation rates were derived from the experimental uncertainty.

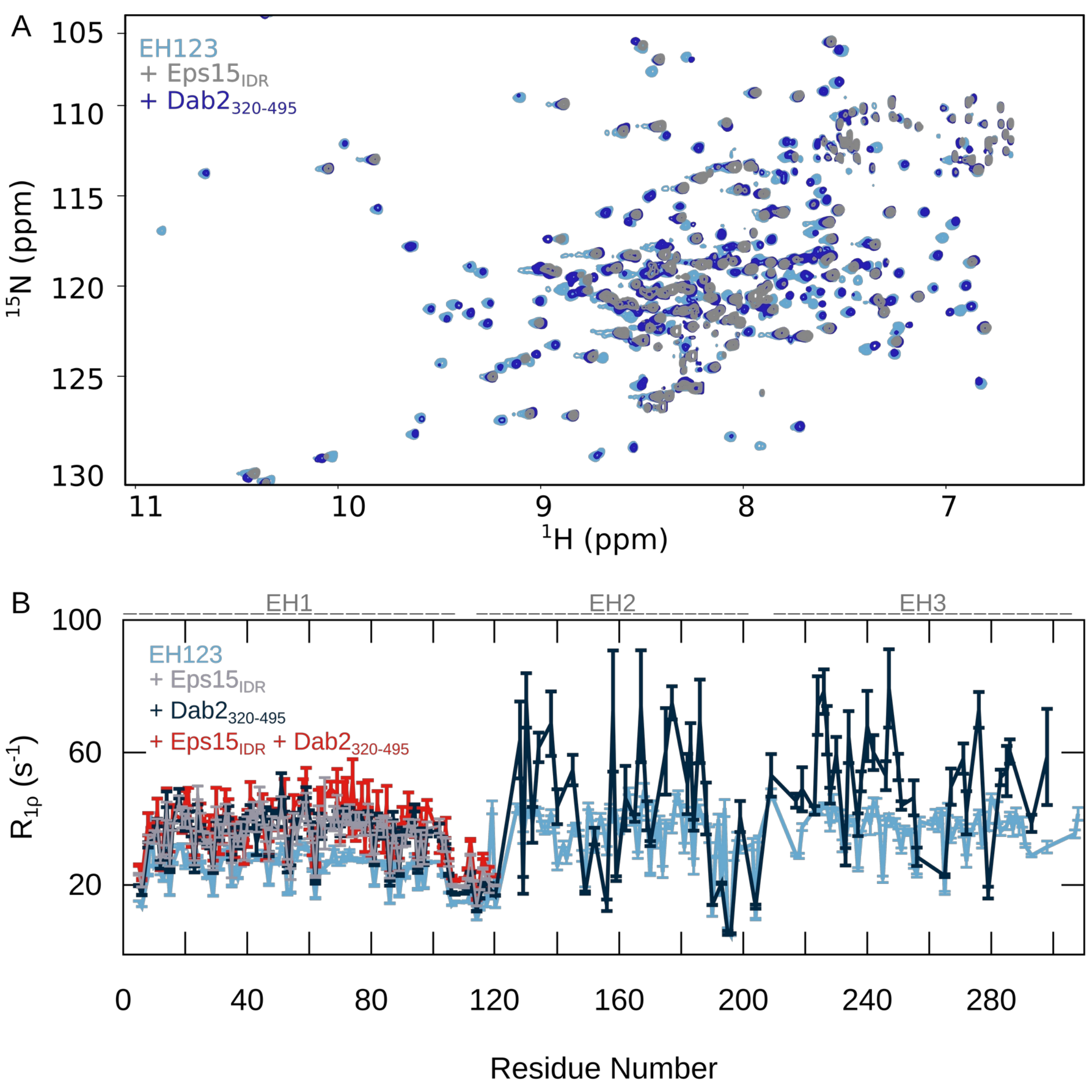

Supplementary Figure 15. Interaction between ^{15}N EH123 and Eps15$_{IDR}$ or Dab2$_{320\text{-}495}$. (A) ^{1}H-^{15}N TROSY-HSQC spectra of 200 µM EH123 alone and in the presence of 200 µM Eps15$_{IDR}$ or 200 µM Dab2$_{320\text{-}495}$. **(B)** ^{15}N $R_{1\rho}$ spin relaxation rates of ^{15}N EH123 in the absence and presence of 100% Eps15$_{IDR}$ and 100% Dab2$_{320\text{-}495}$ and both 100% Dab2$_{320\text{-}495}$ and Eps15$_{IDR}$ at a ^{1}H frequency of 1200 MHz. Only the $R_{1\rho}$ rates within EH1 in the presence of Eps15$_{IDR}$ and Eps15$_{IDR}$ with Dab2$_{320\text{-}495}$ are shown as the peaks of EH2 and EH3 were severely broadened. The parts of EH123 corresponding to EH1, EH2, and EH3 are illustrated above the plots. Color code is indicated in the respective panels. Errors of relaxation rates were derived from the experimental uncertainty.

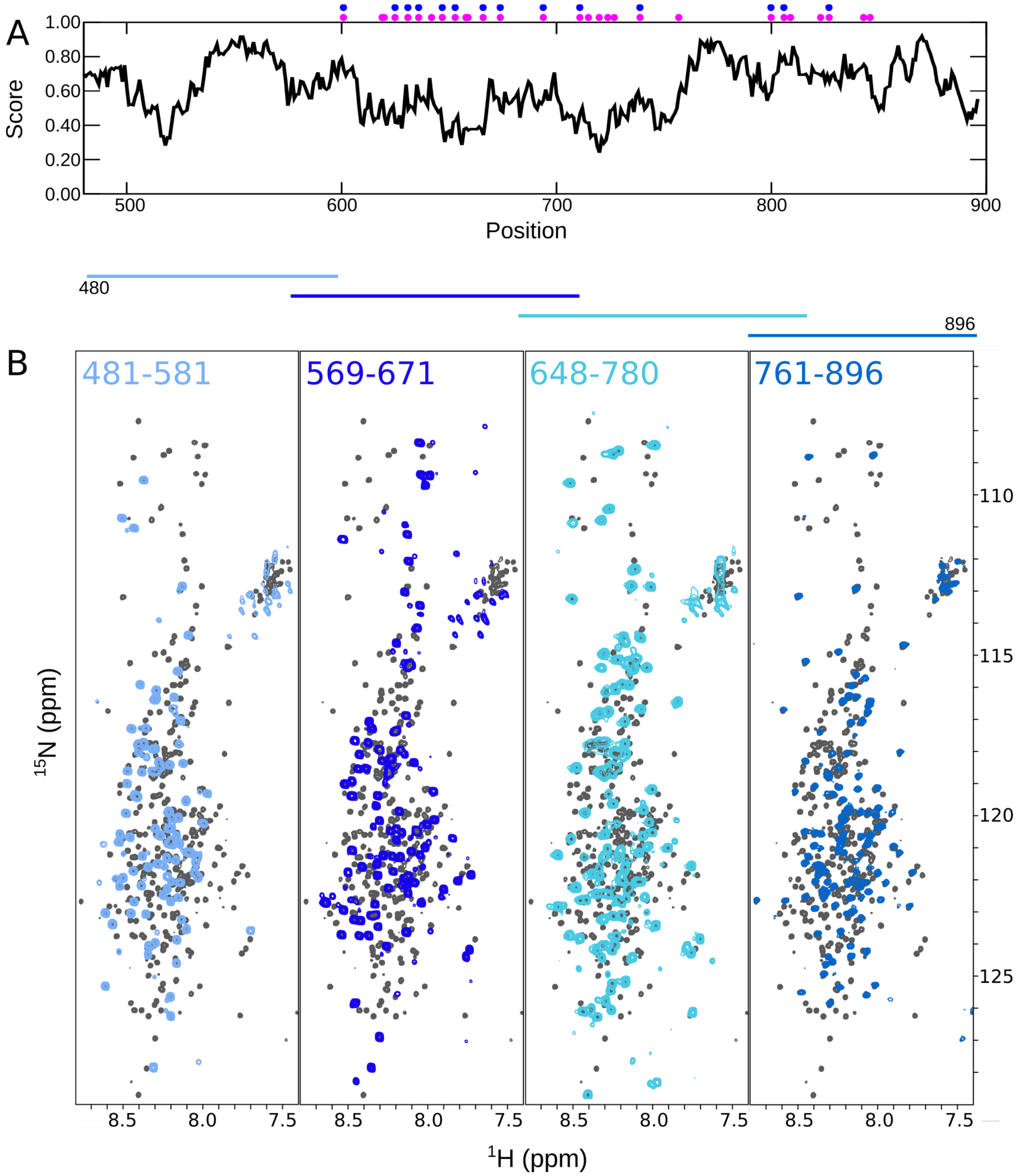

Supplementary Figure 16. Eps15$_{IDR}$ disorder prediction and NMR spectra. (A) IUPred2[23] Disorder prediction of Eps15$_{IDR}$ (black) Larger than 0.5: predicted disordered, Smaller than 0.5: predicted ordered. Filled pink circles denote positions of phenylalanines and filled blue circles denote positions of DPF motifs. **(B)** Superimposition of a ^{1}H-^{15}N HSQC spectrum of Eps15$_{IDR}$ (dark gray) with spectra of the 4 smaller Eps15$_{IDR}$ stretches (different shades of blue).

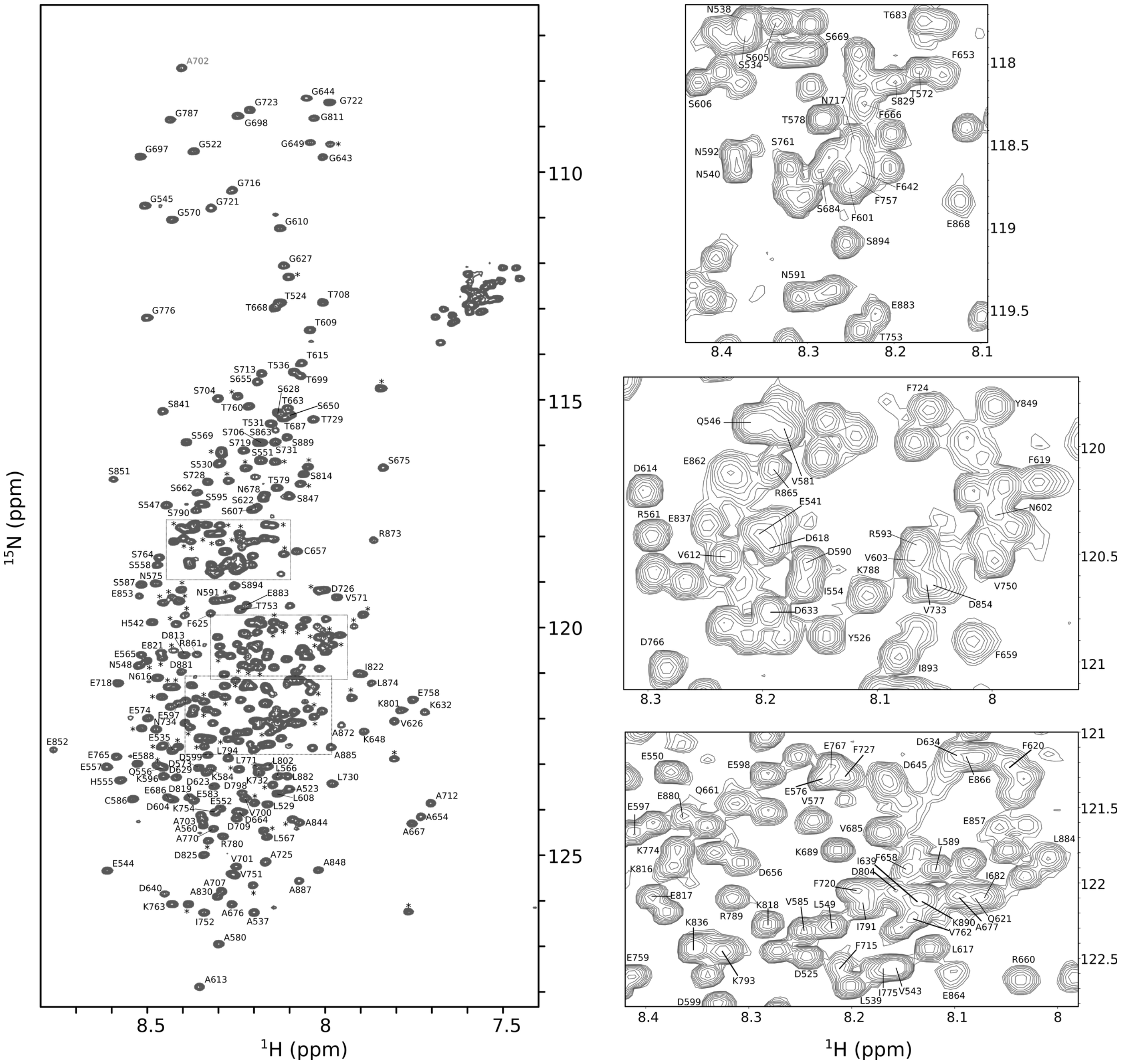

Supplementary Figure 17. Assignment of Eps15$_{IDR}$. ^{1}H-^{15}N HSQC spectrum of Eps15$_{IDR}$ showing the backbone resonance assignments as one letter amino acid code. The regions marked with black boxes are shown as zoom-ins next to the spectrum. A702 appears as a spectrally folded peak. Unassigned peaks are marked with an asterisk in the full spectrum (left). The assignments have been transferred from several shorter Eps15 constructs, of which all non-proline residues have been assigned to 51% for Eps15$_{IDR\ 481\text{-}584}$ (91% starting from residue 521), 86% for Eps15$_{IDR\ 569\text{-}671}$, 54% for Eps15$_{IDR\ 648\text{-}780}$, and 85% for Eps15$_{IDR\ 761\text{-}896}$. Many of the unassigned residues in Eps15$_{IDR\ 648\text{-}780}$ are in the region that overlaps with Eps15$_{IDR\ 569\text{-}671}$, such that the assignment of those residues could be transferred to Eps15$_{IDR}$ full length from the latter construct.

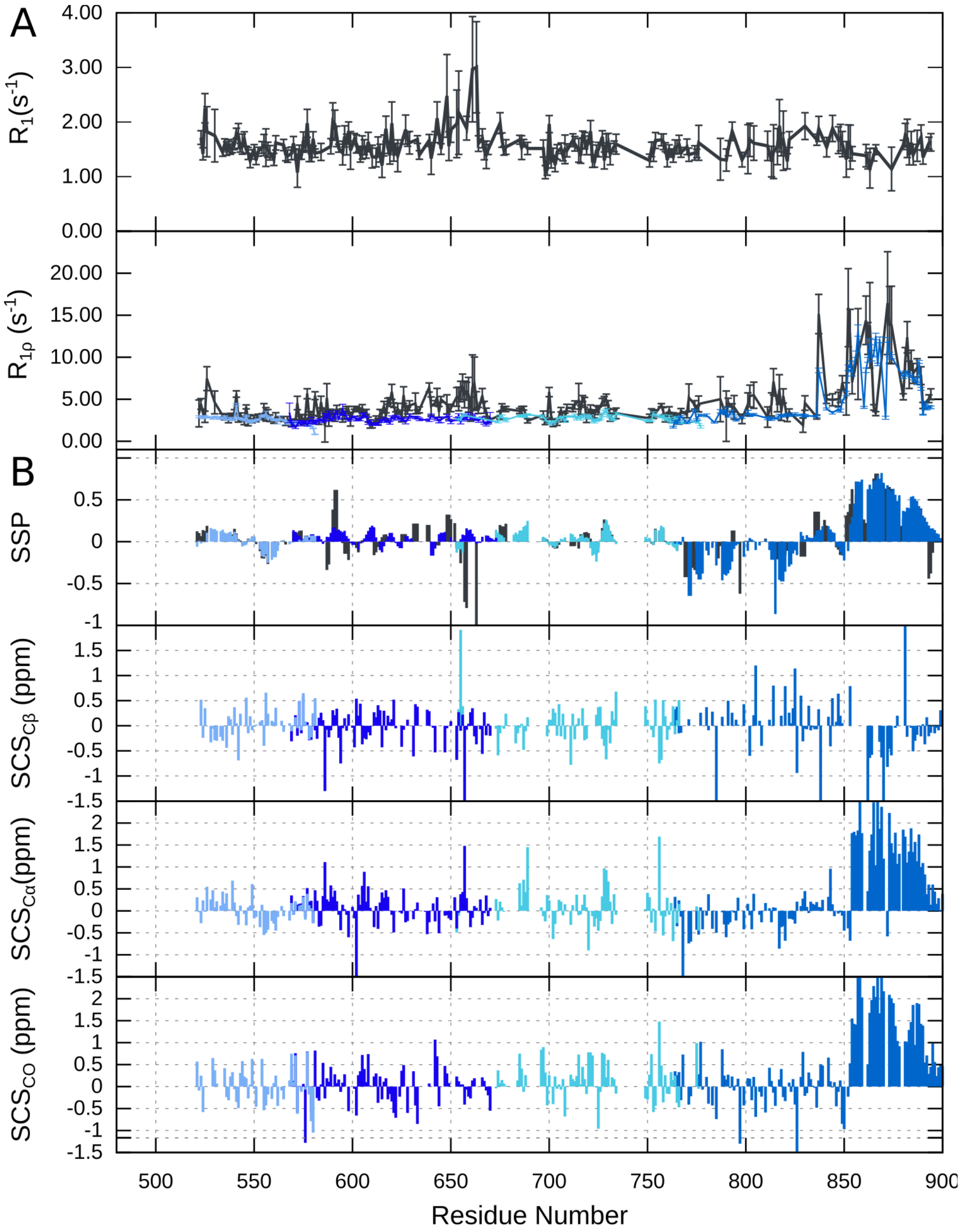

Supplementary Figure 18. Relaxation and secondary chemical shifts of $Eps15_{IDR}$. (A) ^{15}N R_1 and ^{15}N $R_{1\rho}$ spin relaxation of $Eps15_{IDR}$ (black) overlaid with the different smaller stretches of $Eps15_{IDR}$ (different shades of blue). Relaxation rates were recorded at a ^{1}H frequency of 600 MHz. Errors of relaxation rates were derived from the experimental uncertainty. **(B)** SSPs[2] of $Eps15_{IDR}$ (black) as well as SSPs and SCSs of $Eps15_{IDR\ 481\text{-}581}$, $Eps15_{IDR\ 569\text{-}671}$, $Eps15_{IDR\ 648\text{-}780}$, and $Eps15_{IDR\ 761\text{-}896}$ (different shades of blue), showing an alpha helical element in the C-terminus (~residues 850-885). SCSs were calculated with respect to random coil chemical shifts, SSPs were calculated based on Cα and Cβ chemical shifts. A value of 1 reflects a fully formed helix, a value of -1 reflects a fully extended (β-strand) conformation.

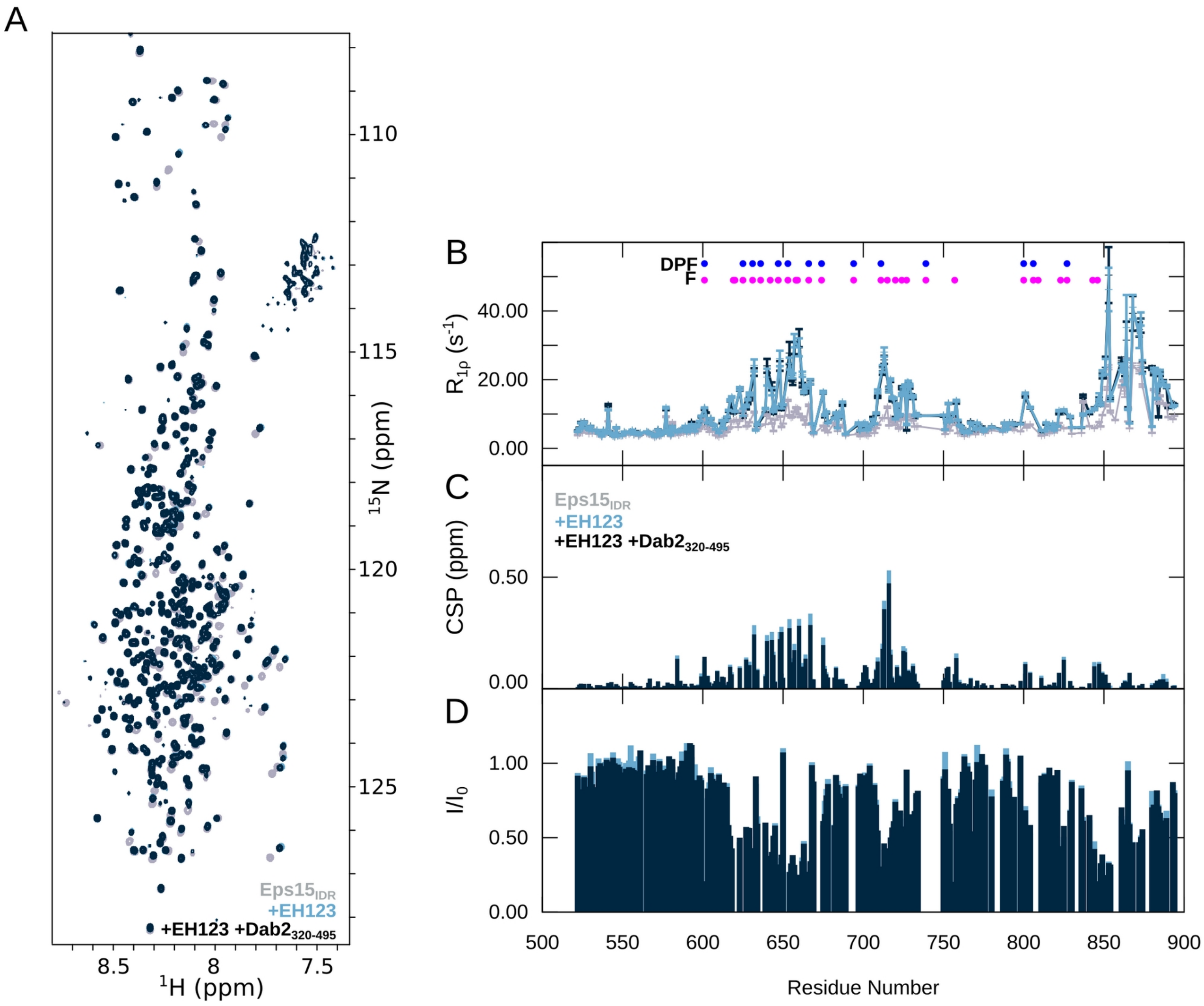

Supplementary Figure 19. Competition experiment between ^{15}N Eps15$_{IDR}$ and Dab2$_{320\text{-}495}$ with EH123. (A) ^{1}H-^{15}N HSQC spectra of 100 µM ^{15}N Eps15$_{IDR}$ alone and in the presence of 100 µM EH123 as well as both 100 µM EH123 and 100 µM Dab2$_{320\text{-}495}$. **(B)** ^{15}N $R_{1\rho}$ relaxation rates recorded at a ^{1}H frequency of 1200 MHz, **(C)** CSPs, and **(D)** intensity ratios of 100 µM Eps15$_{IDR}$ with 100 µM EH123 or both EH123 and Dab2$_{320\text{-}495}$ at 100 µM each. The color codes are displayed in the respective figure panels. Errors of relaxation rates were derived from the experimental uncertainty.

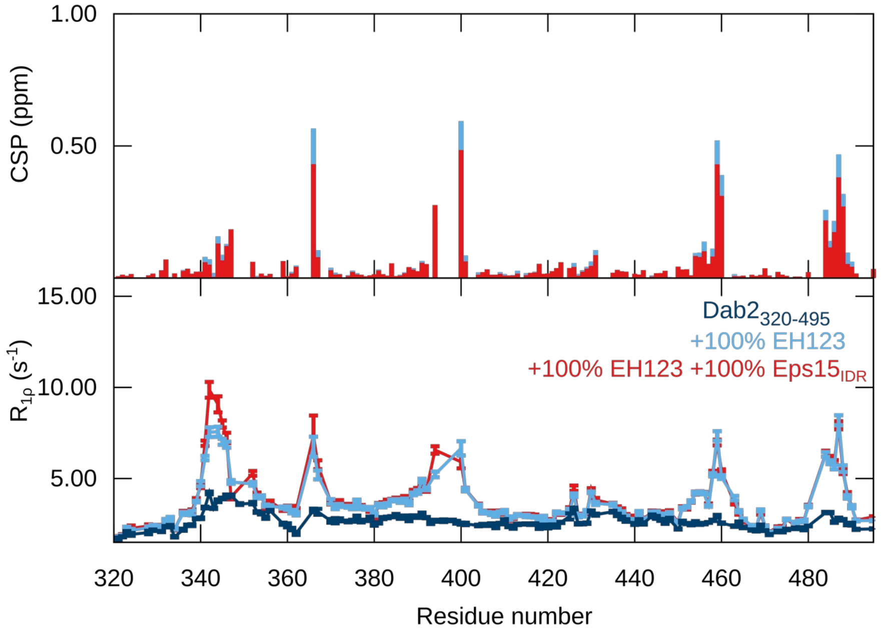

Supplementary Figure 20. Competition experiment between ^{15}N Dab2$_{320-495}$ and Eps15$_{IDR}$ with EH123. CSPs and ^{15}N $R_{1\rho}$ relaxation rates of ^{1}H-^{15}N HSQC of ^{15}N Dab2$_{320-495}$ (100 µM) with EH123 (100 µM) or both 100 µM EH123 and 100 µM Eps15$_{IDR.}$ The relaxation rates have been recorded at a ^{1}H frequency of 600 MHz. The color codes are displayed in the plots. Errors of relaxation rates were derived from the experimental uncertainty.

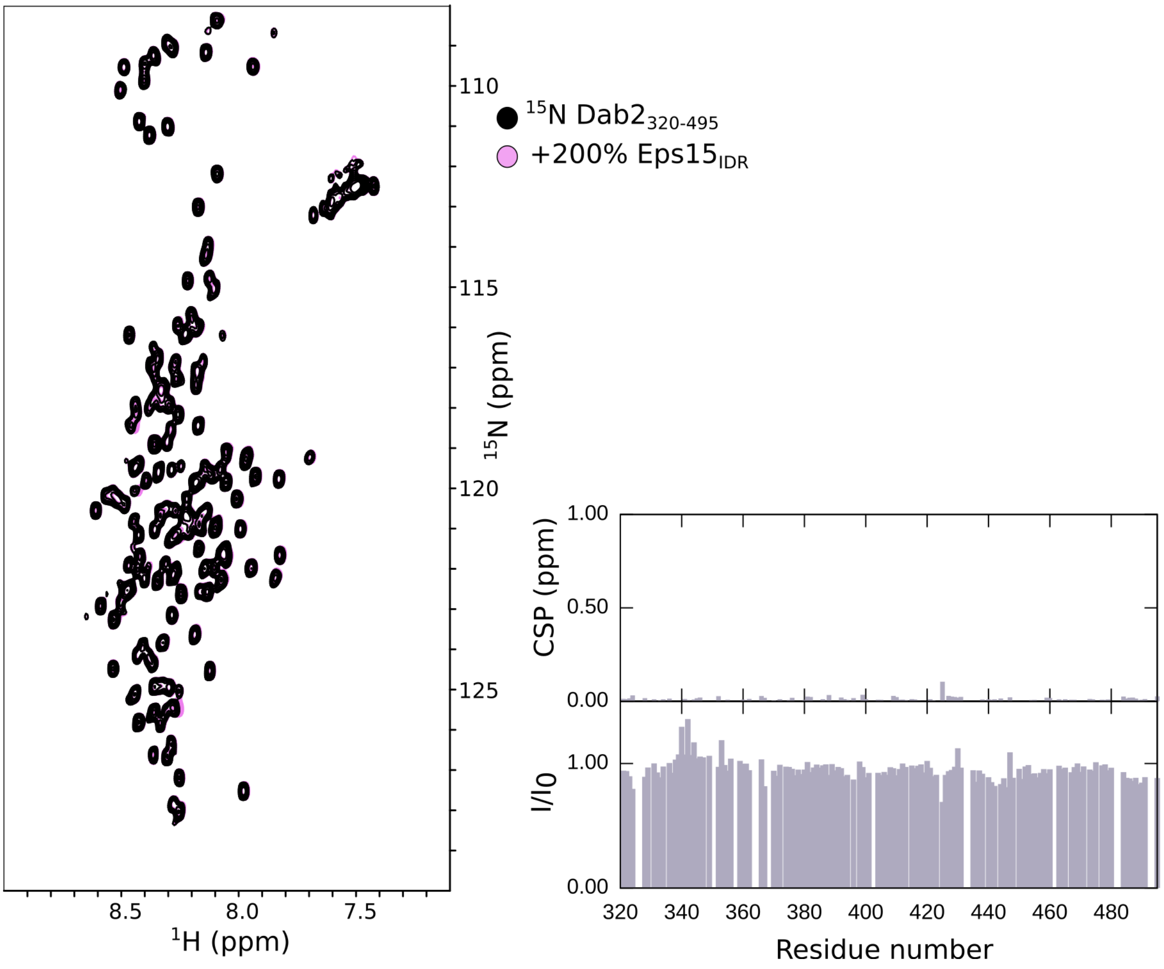

Supplementary Figure 21. Dab2$_{320\text{-}495}$ and Eps15$_{IDR}$ do not interact. Overlay of a spectrum of 100 µM Dab2$_{320\text{-}495}$ (black) with that of 100 µM Dab2$_{320\text{-}495}$ + 200% Eps15$_{IDR}$ (pink), as well as the calculated CSPs and intensity ratios based on the two spectra, confirming that the two IDRs do not interact.

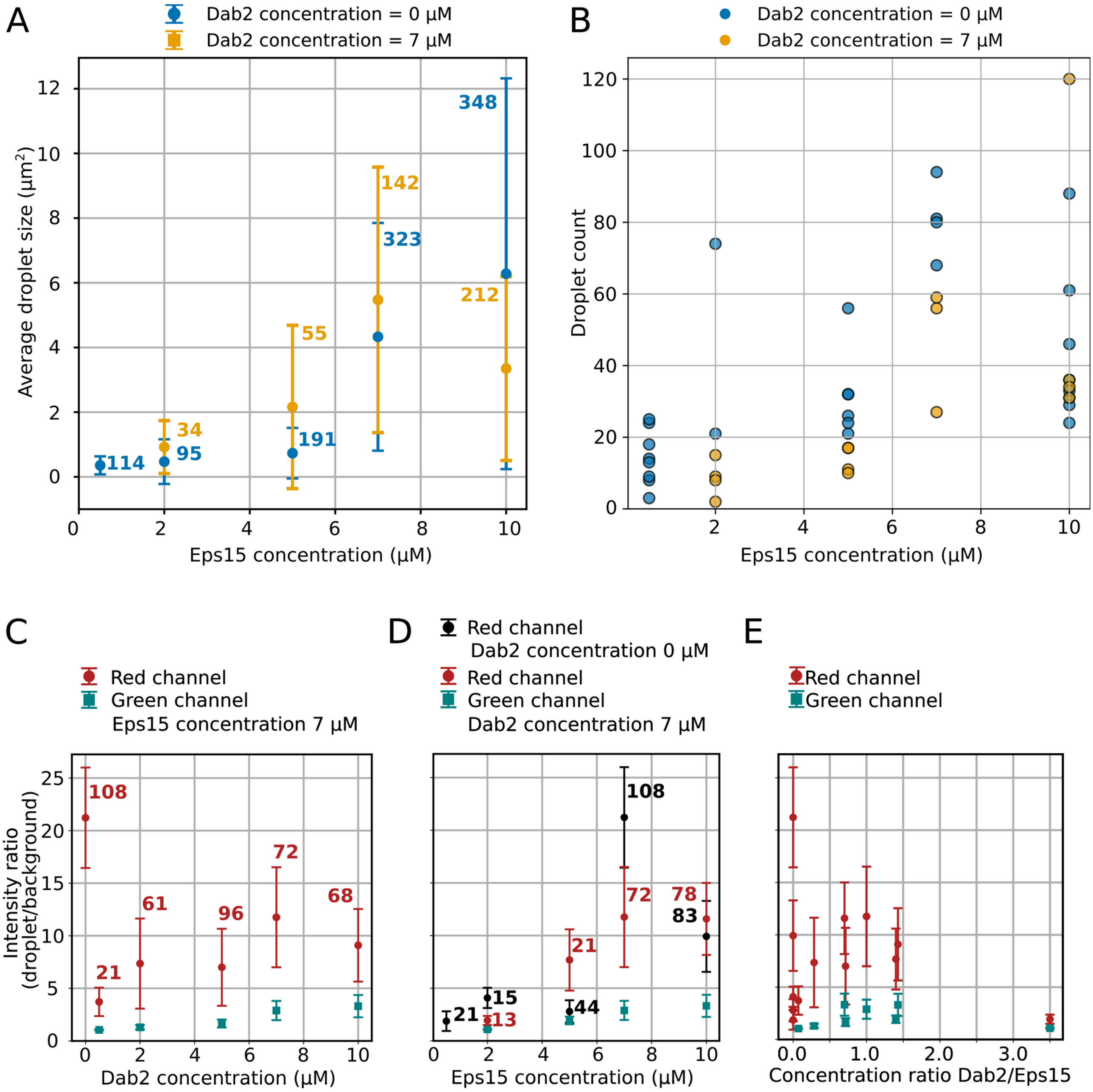

Supplementary Figure 22: The effect of protein concentration on droplet size, droplet count and partitioning of Eps15 and $Dab2_{320\text{-}495}$ into droplets of different composition. (A) The average droplet size plotted against Eps15 concentration in the presence and in the absence of 7 µM $Dab2_{320\text{-}495}$. The number of droplets used to calculate the average is indicated next to each data point. **(B)** The droplet count/image is plotted against Eps15 concentration in the presence and absence of 7 µM $Dab2_{320\text{-}495}$. One point in the plot corresponds to the count in one image. Color codes as indicated in (A). **(C)** Fluorescence intensity ratios inside versus outside the droplets plotted against the $Dab2_{320\text{-}495}$ concentration. Shown are ratios in the green (Dab2) and the red (Eps15) channel. Eps15 was kept at 7 µM through all experiments shown. **(D)** Fluorescence intensity ratios inside versus outside the droplets plotted against the Eps15 concentration. Shown are ratios in the green (Dab2) and the red (Eps15) channel at a $Dab2_{320\text{-}495}$ concentration of 7 µM

and 0 μM. **(E)** Fluorescence intensity ratios inside versus outside the droplets plotted against the concentration ratio of $Dab2_{320\text{-}495}$ versus Eps15 in the red and the green channel. The different points at a ratio of 0 stem from those droplets that were formed with different concentrations of Eps15. The averages in C-E are calculated from the ratios of all analyzed droplets for each condition. The exact number of droplets included in each data point is indicated next to the red channel data points in C-D, and are referring to the data points in E as well. The number of droplets in the red and green channel is the same. Errors are standard deviations of the mean. Source data are provided as a Source Data file.

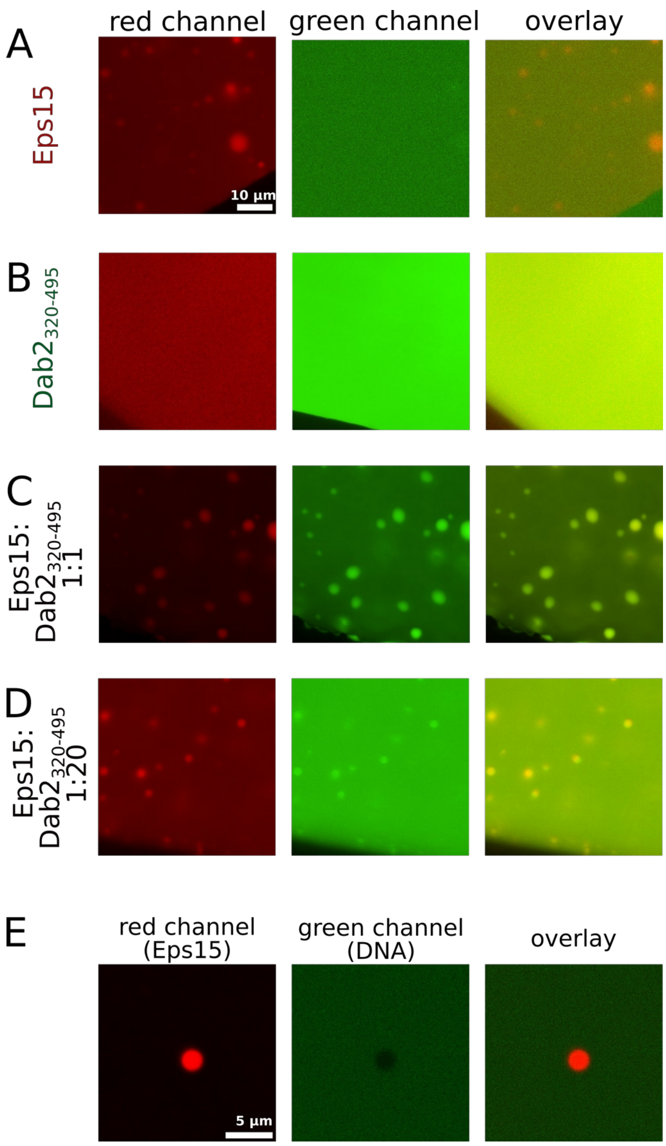

Supplementary Figure 23: Dab2$_{320\text{-}495}$ is recruited into Eps15 droplets at large excess. Microscopy images showing **(A)** droplets of 7 µM Eps15 alone, **(B)** the absence of liquid-liquid phase separation of 7 µM Dab2$_{320\text{-}495}$ alone, and **(C,D)** 7 µM Eps15 and Dab2$_{320\text{-}495}$ in a 1:1 or 1:20 ratio showing recruitment of Dab2 into Eps15 droplets. The scale bar for all images in panels A-D is 10 µm. Images in A-D were acquired on an Epi-fluorescence microscope. **(E)** Exclusion of fluorescently labeled DNA from Eps15 condensates. Droplets of 7 µM Eps15 with 2 µM of Atto488-labeled 38 base pair DNA. The scale bar is 5 µm. Images in (E) were acquired by confocal microscopy.

Dab2 residue number	Kd value from fit to CSPs<br>EH1 (mM)	EH2 (mM)	EH3 (mM)	EH123 (μM)
341	n.d.	1.2±0.6	1.2±0.3	218±14
342	n.d.	1±0.6	2.6±0.3	191±60
366	n.d.	4±3	4±2	160±5
400	0.35±0.05	0.340±0.09	1.39±0.09	104±4
458	n.d.	n.d.	2±1	217±26
460	n.d.	4±3	4±1	253±10
484	n.d.	3±3	4±1	291±7
486	n.d.	10±10	3.0±0.9	378±16

Supplementary Table 1: K_D values fitted for $Dab2_{320\text{-}495}$ binding to the different EH domains based on chemical shift perturbations. In this table, some interactions either do not exist (non-NFP interactions between $Dab2_{320\text{-}495}$ and EH1 for example) or have too weak affinities to be fitted. Those are marked with not determined (n.d.). The data corresponding to the fits are presented in Supplementary Fig. 7A. Shown are the results of fit corresponding to a two state binding model as described in the Methods section with their respective fitting errors.

Sample	Parameters from CPMG fit k_{ex} (s^{-1})	Percentage bound (%)	Calculated kd (μM)
100μM 15N $Dab2_{320\text{-}495}$ +10μM EH2	149±13	3.3±0.3	196

Supplementary Table 2: K_D values fitted for $Dab2_{320\text{-}495}$ binding to EH2 assessed by CPMG relaxation dispersion. Shown are the exchange rate (k_{ex}), the percentage of bound $Dab2_{320\text{-}495}$, and the K_D value calculated from the percentage bound and the protein concentrations used in the experiment (see also Fig. Supplementary Fig. 7B) with the corresponding fitting errors. We did not propagate the fitting errors to the calculated K_D, because the error in concentration determination likely significantly exceeds the fitting error.

Supplementary References

1. Zhang, H., Neal, S. & Wishart, D. S. RefDB: a database of uniformly referenced protein chemical shifts. *J Biomol NMR* **25**, 173–195 (2003).
2. Marsh, J. A., Singh, V. K., Jia, Z. & Forman-Kay, J. D. Sensitivity of secondary structure propensities to sequence differences between α- and γ-synuclein: Implications for fibrillation. *Protein Sci* **15**, 2795–2804 (2006).
3. Kragelj, J. *et al.* Structure and dynamics of the MKK7-JNK signaling complex. *Proc Natl Acad Sci U S A* **112**, 3409–3414 (2015).
4. Whitehead, B., Tessari, M., Carotenuto, A., van Bergen en Henegouwen, P. M. P. & Vuister, G. W. The EH1 Domain of Eps15 Is Structurally Classified as a Member of the S100 Subclass of EF-Hand-Containing Proteins. *Biochemistry* **38**, 11271–11277 (1999).
5. de Beer, T. *et al.* Molecular mechanism of NPF recognition by EH domains. *Nat Struct Biol* **7**, 1018–1022 (2000).
6. Enmon, J. L., de Beer, T. & Overduin, M. Solution Structure of Eps15's Third EH Domain Reveals Coincident Phe−Trp and Asn−Pro−Phe Binding Sites,. *Biochemistry* **39**, 4309–4319 (2000).
